# Comprehensive Metabolic Profiling and Genome-wide Analysis Reveal Therapeutic Modalities for Hepatocellular Carcinoma

**DOI:** 10.34133/research.0036

**Published:** 2023-01-13

**Authors:** Feng Qi, Jia Li, Zhuoran Qi, Jian Zhang, Bin Zhou, Biwei Yang, Wenxing Qin, Wenguo Cui, Jinglin Xia

**Affiliations:** ^1^ Department of Oncology, Ruijin Hospital, Shanghai Jiao Tong University School of Medicine, Shanghai 200000, China.; ^2^ National Medical Center and National Clinical Research Center for Interventional Medicine, Liver Cancer Institute, Zhongshan Hospital, Fudan University, 180 Fenglin Road, Shanghai 200032, China.; ^3^ Department of Hepatic Surgery VI, Eastern Hepatobiliary Surgery Hospital, Second Military Medical University, Shanghai 200433, China.; ^4^ Phase I Clinical Trial Center, Fudan University Shanghai Cancer Center; Department of Oncology, Shanghai Medical College, Fudan University, No. 270, Dong’an Road, Shanghai 200032, China.; ^5^ Department of Orthopaedics, Shanghai Key Laboratory for Prevention and Treatment of Bone and Joint Diseases, Shanghai Institute of Traumatology and Orthopaedics, Ruijin Hospital, Shanghai Jiao Tong University School of Medicine, Shanghai 200000, China.; ^6^ Key Laboratory of Diagnosis and Treatment of Severe Hepato-Pancreatic Diseases of Zhejiang Province, The First Affiliated Hospital of Wenzhou Medical University, Wenzhou 325000, China.

## Abstract

Understanding the details of metabolic reprogramming in hepatocellular carcinoma (HCC) is critical to improve stratification for therapy. Both multiomics analysis and cross-cohort validation were performed to investigate the metabolic dysregulation of 562 HCC patients from 4 cohorts. On the basis of the identified dynamic network biomarkers, 227 substantial metabolic genes were identified and a total of 343 HCC patients were classified into 4 heterogeneous metabolic clusters with distinct metabolic characteristics: cluster 1, the pyruvate subtype, associated with upregulated pyruvate metabolism; cluster 2, the amino acid subtype, with dysregulated amino acid metabolism as the reference; cluster 3, the mixed subtype, in which lipid metabolism, amino acid metabolism, and glycan metabolism are dysregulated; and cluster 4, the glycolytic subtype, associated with the dysregulated carbohydrate metabolism. These 4 clusters showed distinct prognoses, clinical characteristics and immune cell infiltrations, which was further validated by genomic alterations, transcriptomics, metabolomics, and immune cell profiles in the other 3 independent cohorts. Besides, the sensitivity of different clusters to metabolic inhibitors varied depending on their metabolic features. Importantly, cluster 2 is rich in immune cells in tumor tissues, especially programmed cell death protein 1 (PD-1)-expressing cells, which may be due to the tryptophan metabolism disorders, and potentially benefiting more from PD-1 treatment. In conclusion, our results suggest the metabolic heterogeneity of HCC and make it possible to treat HCC patients precisely and effectively on specific metabolic characteristics.

## Introduction

Hepatocellular carcinoma (HCC) remains one of the most common malignant tumors and the second leading cause of cancer-related mortality in the world [1–3]. The development of HCC is a complex biological process that involves the interplay of various factors, including genetic and epigenetic alterations, viral infection [4], altered cellular microenvironment, and various immune cells [5]. The highly heterogeneity of HCC seriously restricts early diagnosis and the research of HCC molecular mechanism as well as the exploration of precision treatment. Therefore, a better understanding of the pathogenesis of the driver of HCC is crucial. Although many genes and their expression changes can influence the progression of HCC patients, it is difficult to make an early diagnosis. HCC progression can be divided into 3 stages: predisease state, blast crisis, and advanced disease state. Studies found that there was a phase transition following the blast crisis, leading to an irreversible change in diseases [6]. Since the blast crisis occurs soon after the predisease state, there is no significant difference between the predisease state and the blast crisis. "Traditional" molecular biomarkers cannot identify HCC patients who are in a blast crisis. Nowadays, dynamic analysis methods are becoming more and more important, detecting a significant difference between predisease state and blast crisis, which makes analysis at blast crisis possible and can be used to screen dynamic network biomarkers (DNBs) [7].

In the dynamic changes of HCC development, metabolic reprogramming, a typical hallmark of cancer, plays an important role in HCC diagnosis, prognosis, and treatment [8,9]. However, recent metabolic reprogramming studies only focus on the advanced disease state of tumors but lack attention on blast crisis. Similarly, metabolic drugs are usually delivered at the advanced disease state without any indication of metabolic dependence in previous failed trials [10,11]. The wrong time point may be the cause of failure while studies on dynamic changes at blast crisis are required.

In past years, considerable effort has been expended to divide HCC into several molecular subtypes with different static mutation profiles and genomic alterations on the advanced disease state [12]. However, it is powerless to analyze the complex dynamic changes of HCC from a single static perspective on the advanced disease state only. A study focusing on the systemic dynamic analysis of HCC at blast crisis from the viewpoint of multiomics analysis is urgently needed.

On the basis of the significant role of metabolic reprogramming and multiomics analysis demand at blast crisis, we proceed a comprehensive assessment of the overall dynamic metabolic profile of HCC. We extract 227 DNBs of HCC at blast crisis and divided them into 4 clusters based on their expression trends. Clusters with different heterogeneity have different genetic alterations, metabolic characteristics, immune microenvironment, prognosis, and sensitivity to targeted and immune drugs.

## Result

### Metabolic dysregulation occurred in HCCs

**Fig. 1.**
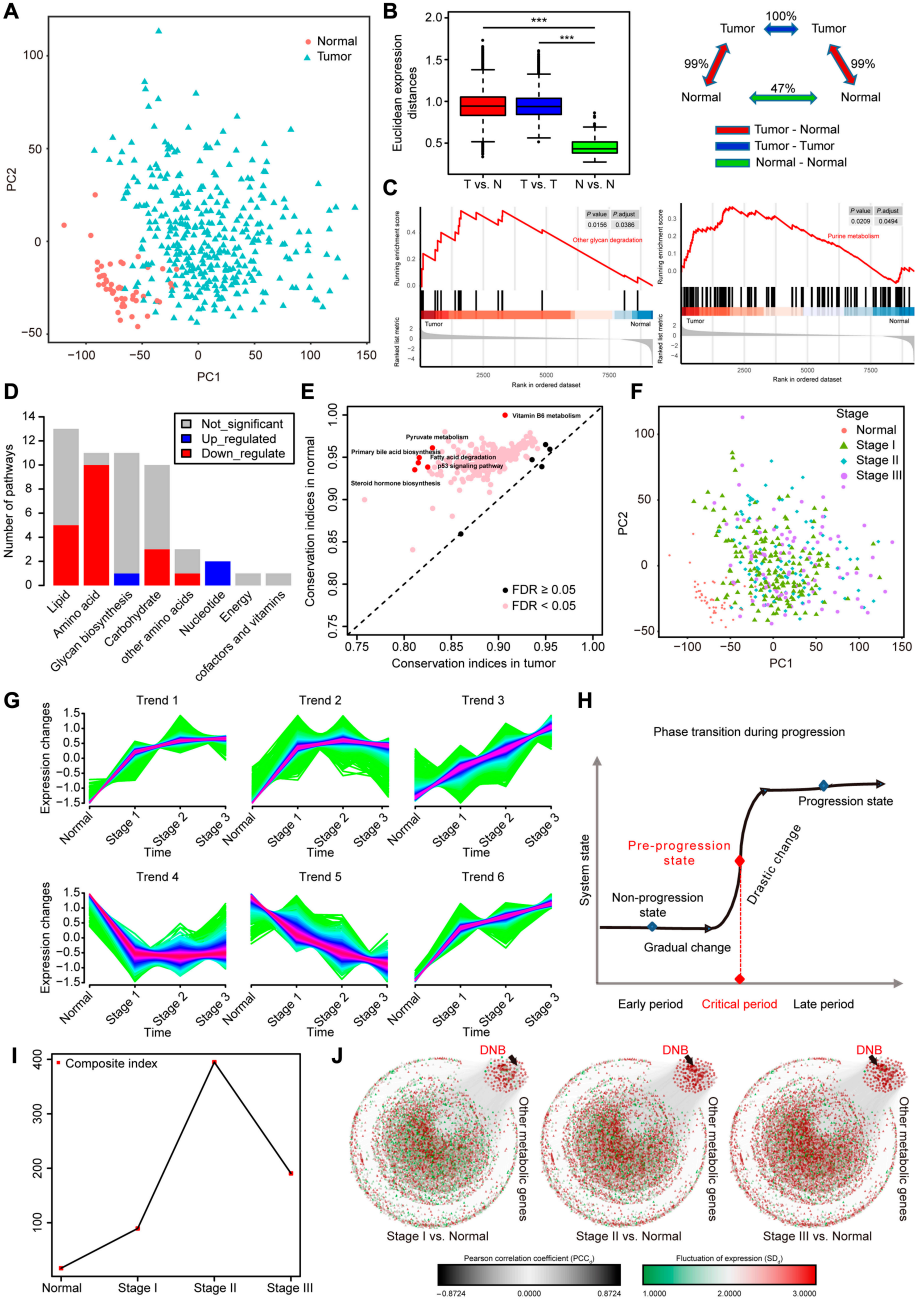
Metabolic dysregulation and dynamic changes in HCCs. (A) PCA of HCC tumor tissues (*n* = 343) and paired normal tissues (*n* = 49) in the TCGA-HCC cohort. (B) Global differences in metabolic gene expression between HCC tumors and normal tissues in the TCGA-HCC cohort. The Euclidean expression distances were calculated between tumors and normal tissues (red), within samples of tumor tissues (blue), and within samples of normal tissues (green). The inset summarizes the average distances between pairs of tissues as a percentage of the average distance between tumors and normal tissues. (Level of significance: ***P < 0.001.) (C) A representative GSEA plot showing significantly dysregulated glycan degradation (left) and purine metabolism (right) in the tumors versus normal tissues in the TCGA-HCC cohort. (D) The number of metabolic pathways that were significantly dysregulated (FDR < 0.05) in the tumors versus normal tissues in the TCGA-HCC cohort among each of 8 metabolic categories. The X-axis represents metabolic categories where metabolic pathways were classified according to the KEGG database. The Y-axis represents the number of pathways classified into corresponding category. (E) Comparison of the RCI of metabolic pathways between tumors and normal tissues in the TCGA-HCC cohort. FDR < 0.05 indicates differentially regulated pathways between tumors and normal tissues in the TCGA-HCC cohort. (F) PCA of HCC tumors at different stage and paired normal tissues in the TCGA-HCC cohort. (G) The series of diagrams depicts the dynamic changes pattern of 9193 metabolic genes during different stages in the TCGA-HCC cohort, using Mfuzz. (H) A schematic diagram illustrates a stage transition during HCC progression. The critical period after the early period changes the state of the biological system qualitatively and thus plays a key role in biological processes. (I) The graph illustrates that the critical transition occurs at Stage II of HCC, according to CIs over all time points in gene expression profiling. (J) The series of networks shows that the 3 criteria of DNB were satisfied at Stage II of HCC from dynamic changes in gene expression.

To explore HCC metabolic reprogramming characters, we obtained a total of 9,193 human metabolic genes which were assigned to 344 metabolic pathways in the Kyoto Encyclopedia of Genes and Genomes (KEGG) database. In the The Cancer Genome Atlas (TCGA)-HCC cohort, principal component analysis (PCA) showed that metabolic gene expressions of HCC were different from normal tissues (Fig. 1A). We then calculated Euclidean distance to investigate the global variance in metabolic gene expression between and within HCC tissues and their corresponding normal tissues. The expression distances between HCC and normal tissues or within HCC tissues were significantly larger than that within normal tissues, indicating the metabolic divergence within HCC tissues (Fig. 1B).

Then, we investigated whether the selected metabolic genes could explain the dysregulated metabolic functions in HCC. Gene set enrichment analysis (GSEA) revealed that, compared with normal tissues, a total of 22 metabolic pathways were significantly dysregulated in HCC tissues (false discovery rate [FDR] < 0.05, 3 pathways upregulated and 19 pathways downregulated). The upregulated pathways involved in glycan biosynthesis and nucleotide, such as other glycan degradation and purine metabolism pathways, whereas the downregulated pathways mainly included lipid, amino acid, and carbohydrate metabolisms (Fig. 1C and D). Besides, conservation differences of metabolic pathways between tumor and normal tissues were quantified by using differential rank conservation (DIRAC) analysis (Fig. 1E). The majority of metabolic pathways had significantly lower rank conservation indices (RCIs) in HCC tumors (FDR < 0.05), indicating the higher variability and deregulation of metabolic pathways at the transcriptional level in HCC tumors. These results showed that there are noteworthy metabolic heterogeneity and dysregulation in HCC tumors.

However, there was no clear difference in metabolic gene expression among 3 stages of the TCGA-HCC cohort (Fig. 1F), suggesting that traditional differential gene analysis may not be suitable for pinpointing the critical state. To characterize the dynamic changes in terms of gene expression during different tumor stages, we clustered these metabolic genes into 6 clusters (Trend 1 to Trend 6) via Mfuzz method [7] (Fig. 1G). Genes in Trends 1, 3, and 6 were significantly upregulated from the normal group to the Stage III group, while genes in Trend 5 were downregulated. Genes in Trends 2 and 4 were upregulated or downregulated only from the normal group to the Stage I group, respectively. These results suggested that disease progression was not gradual and monotonic, but nonlinear and drastic at certain points [13]. The significant changes in metabolic gene expression from normal to stage III indicated the possibility of predicting tipping point. Different from the traditional static biomarkers with differential expression used previously, DNB members are dynamic biomarkers for a generally irreversible transition [6,13] (Fig. 1H). According to 3 criteria, we identified in total 227 DNBs (see Materials and Methods, Fig. 1I, and Fig. S1A to C) when the system stage gradually achieved a tipping point. The distribution of criticality index (CI) values suggested a strong signal of the critical state at Stage II (Fig. 1I). On the basis of protein-protein interactions, we displayed the dynamics of DNBs as a network during the progression of HCC, where nodes and links were respectively weighted by SDs of gene expressions and Pearson correlation coefficients of pairwise gene expressions in a given stage versus those in normal group (Fig. 1J). Compared with the whole molecular network, we found that metabolic DNBs could signal the tipping point at Stage II, indeed.

### Metabolic clusters of HCC based on DNBs

**Fig. 2.**
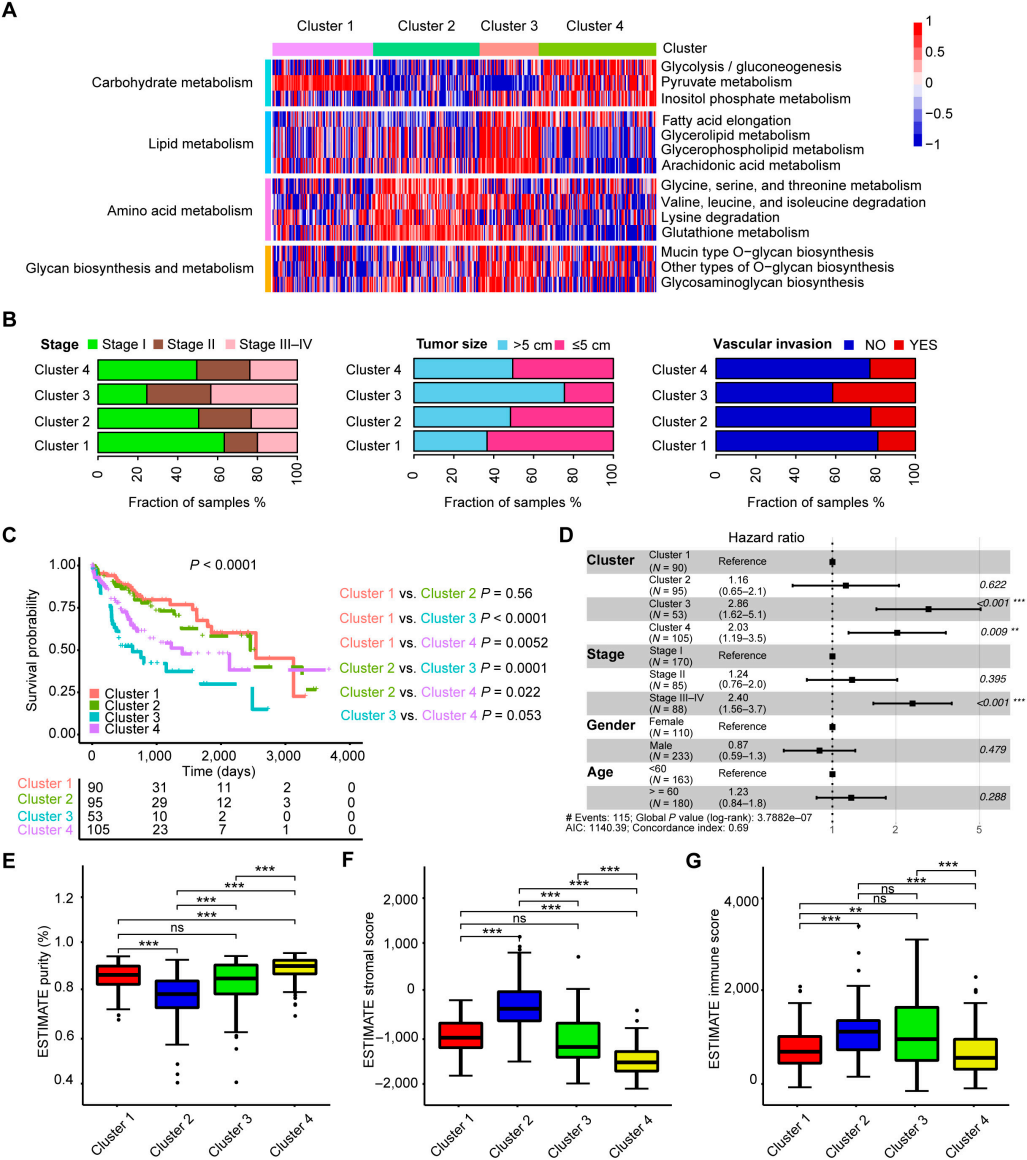
Metabolic clusters of HCC based on DNBs. (A) The clustering results based on the enrichment scores of 227 DNBs of HCC tumors in the TCGA-HCC cohort. Heatmap shows normalized enrichment scores of the 4 metabolic clusters. (B) Bar plots show the distribution of HCC clinical characteristics, including disease stage, tumor size, and condition of vascular invasion among the 4 metabolic clusters. (C) Kaplan–Meier curves of survival among the 4 metabolic clusters in the TCGA-HCC cohort. Log-rank test and BH method for adjusting *P* value. Among all clusters, *P* < 0.0001; cluster 1 versus cluster 2, *P* = 0.56; cluster 1 versus cluster 3, *P* < 0.0001; cluster 1 versus cluster 4, *P* = 0.0052; cluster 2 versus cluster 3, *P* = 0.0001; cluster 2 versus cluster 4, *P* = 0.022; cluster 3 versus cluster 4, *P* = 0.053. (D) Forest plot of multivariate Cox regression analysis for survival adjusting for disease stage, gender, age, and metabolic clusters in the TCGA-HCC cohort. The hazard ratios are shown with 95% confidence intervals. ***P < 0.001; **P < 0.01; ns, *P* > 0.05. (E to G) Boxplots show the distribution of tumor purity (E), stromal score (F), and immune score (G) among the 4 clusters. Tukey’s post hoc test. (Level of significance: ***P < 0.001; **P < 0.01; ns, *P* > 0.05.)

To reveal the metabolic heterogeneity of HCC tumors, we estimated the enrichment scores of 155 metabolic pathways enriched from 227 DNBs in the TCGA-HCC cohort through gene set variation analysis (GSVA) (Table S1). On the basis of enrichment scores of 155 metabolic pathways, 343 HCC tumor samples were clustered into 4 heterogeneous clusters via unsupervised *k*-means clustering (Fig. 2A). The optimal cluster number was identified through consensus clustering and NbClust testing, and Silhouette analysis demonstrated the stability of clustering results with k as 4 (Fig. S2, A to C). We found that cluster 1 and cluster 2 were less metabolically active than cluster 3 and cluster 4, and the clear metabolic changes were observed in cluster 3 (Fig. 2A). Despite the complexity of metabolic genes in each cluster, we characterized each cluster in detail according to their metabolic expression differences. Cluster 1, designated the pyruvate subtype, was characterized by upregulation of the pyruvate metabolism pathway. Cluster 2, designated the amino acid subtype, was characterized by the dysregulation of amino acid metabolism pathways, mainly including glutathione metabolism. Cluster 3, designated the mix-metabolic type, was characterized by the dysregulation of lipid metabolism, amino acid metabolism, and glycan biosynthesis and metabolism. Cluster 4, thereafter designated the glycolytic subtype, was characterized by remarkable dysregulation of carbohydrate pathways, including glycolysis, pyruvate metabolism, and inositol phosphate metabolism. We then investigated the overlap between the clusters and HCC clinical characters (Fig. 2B and Table S2). Cluster 1 had a higher rate of early-stage patients, smaller tumor sizes, and less vascular invasion, whereas cluster 3 had a higher rate of terminal-stage patients, bigger tumor size, and more vascular invasion. Moreover, cluster 1 and cluster 2 showed significantly better survival than cluster 3 and cluster 4, while cluster 3 showed worse survival than the other 3 clusters (Fig. 2C). Multivariate Cox proportional hazard models also revealed that cluster 3 independently predicted a worse prognostic in HCC (Fig. 2D). We found that the tumor purity, tumor-stroma score, and immune score were rather different among the 4 clusters (Fig. 2E to G). In sum, our results demonstrated that the metabolic heterogeneity of HCCs fell into 4 metabolic clusters, but they could not be fully explained by transcriptome-based subtyping.

### Genomic alterations of metabolic clusters in HCCs

**Fig. 3.**
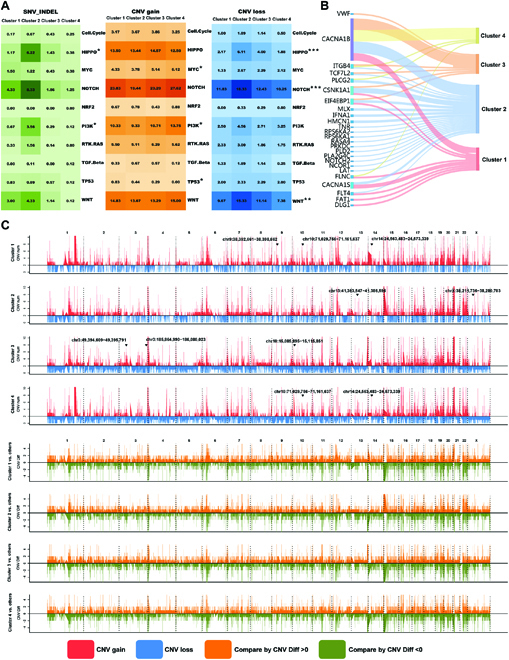
Genomic alterations of metabolic clusters in HCCs. (A) Genomic alterations in 10 oncogenic pathways were compared between all possible pairs of the 4 metabolic clusters in the ZS-SEQ-HCC cohort. The color in the box represents different types of genomic alterations (green, SNV_INDEL; orange, CNV gain; blue, CNV loss), and the color saturation represents the mutation frequency. The pathways with significant differences in mutation frequency in at least 1 of pairwise comparison among 4 clusters were labeled with asterisk. (Mann–Whitney test; level of significance: ***P < 0.001; **P < 0.01; *P < 0.05.) (B) Sankey diagram for the mutation frequency of genes that showed a significant difference (*P* < 0.05) in the comparison between all possible pairs among the 4 metabolic clusters in the ZS-SEQ-HCC cohort. (C) Comparison of the somatic CNVs between 1 to remaining clusters among the 4 metabolic clusters in the ZS-SEQ-HCC cohort. The upper plot shows the number of CNV gain (red) and CNV loss (blue) of each gene in each cluster. The lower plot shows difference of CNV numbers (represented as CNF Diff) between 1 to remaining clusters. Orange, compared by CNV Diff > 0; green, compared by CNV Diff < 0.

In this study, we used the genomics data of HCC samples from the ZS-SEQ-HCC cohort with both whole-exome sequencing (WES) and RNA-sequencing data to explore genomic alterations among the 4 metabolic clusters. We firstly used GSVA to obtain the enrichment scores of 155 metabolic pathways mentioned before in 30 HCC samples of the ZS-SEQ-HCC cohort. By using the nearest shrunken centroids (NSC) method (see Materials and Methods), there were 6, 9, 7, and 8 HCC samples classified into cluster 1, cluster 2, cluster 3, and cluster 4, respectively (Fig. S3A and B). Cluster 2 had a relatively higher copy-number variation (CNV) burden and mutation burden among the 4 clusters (Fig. S3C and D). We further investigated the frequency of mutations and CNVs in the ZS-SEQ-HCC cohort. Ten oncogenic signaling pathways [14] were used to explore the genomic characteristics for each cluster. We found that cluster 2 had a significantly higher frequency of single-nucleotide variant (SNV)-INDELs in the HIPPO and phosphatidylinositol 3-kinase (PI3K) pathways (Fig. 3A and Table S3). In terms of CNV gain and CNV loss, we found there were significant differences in mutation number of some oncogenic pathways among clusters, such as CNV gain of the MYC, PI3K, and TP53 pathways and CNV loss of the HIPPO, NOTCH, and WNT pathways (Fig. 3A). When exploring cluster-specific mutated genes, we found that cluster 2 had a higher frequency of mutations among the HIPPO pathway members (HMCN1 and TCF7L2) and PI3K pathway members (EIF4EBP1 and IFNA1) (Fig. 3B and Table S3). To explain the metabolic characteristics and heterogeneity of 4 clusters, we moved attention to the mutation events in metabolic genes (Fig. 3C). Our results demonstrated that cluster 1 and cluster 4 had more frequent somatic copy-number alterations in glycolytic genes, including HK1 (chr10:71,029,756-71,161,637), ALDH1B1 (chr9:38,392,661-38,398,662), and PCK2 (chr14:24,563,483-24,573,339), while cluster 2 had more frequent somatic copy-number alterations in amino acid metabolism genes, including OTC (chr10:71,029,756-71,161,637) and SLC25A1 (chr9:38,392,661-38,398,662), and cluster 3 had more frequent copy-number alterations in lipogenic genes, including OLAH (chr10:15,085,895-15,115,851), GPX1 (chr3:49,394,609-49,395,791), and DGKG (chr3:185,864,990-186,080,023). Besides, the CNV status of these genes in the mentioned genomic region were positively associated with their mRNA expression (Fig. S4A to C). Similar to the ZS-SEQ-HCC cohort, we also found genomic changes in the TCGA-HCC cohort, which showed the significant differences in fraction of genome altered (https://www.cbioportal.org/study/summary?id=lihc_tcga_pan_can_atlas_2018) and tumor mutation burden among the 4 clusters, especially cluster 2 (Fig. S5). Overall, our results indicated that genomic alterations in specific chromosomal regions might mediate the metabolic reprogramming and heterogeneity in HCC.

### Metabolic clusters show distinct metabolic characteristics

**Fig. 4.**
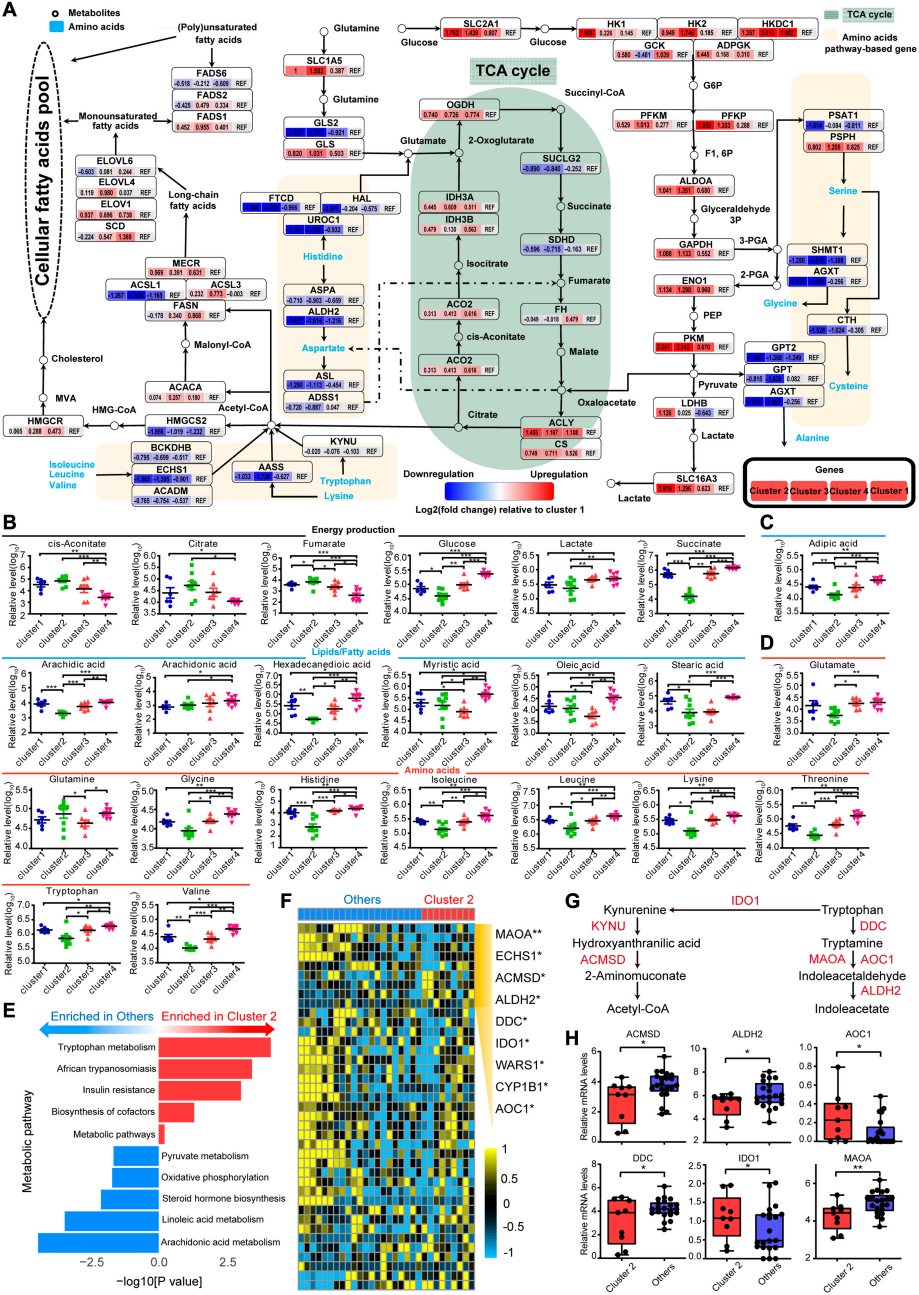
Metabolic clusters of HCC show distinct metabolic characteristics. (A) Diagram shows the glycolysis, tricarboxylic acid (TCA) cycle, lipid metabolism, and amino acid metabolism pathways enriched by differential metabolites in the ZS-SEQ-HCC cohort. The expression alteration of genes involved in the plotted pathways are depicted as log2 fold change, with fold change as the ratio of average mRNA expression of genes in each cluster versus those in cluster 1. Red, upregulated genes compared with cluster 1; blue, downregulated genes compared with cluster 1. (B to D) Relative levels of metabolites, involved in (B) carbohydrate metabolism, (C) lipid metabolism, and (D) amino acid metabolism, that were differentially expressed among the 4 metabolic clusters in the ZS-SEQ-HCC cohort. Tukey’s post hoc test. (E) Bar plot shows metabolic pathway enriched by differential metabolites between cluster 2 and other clusters in the ZS-SEQ-HCC cohort. Red, upregulated pathways in cluster 2; blue, downregulated pathways in cluster 2. (F) Heatmap shows relative mRNA expression of genes related to tryptophan metabolism in cluster 2 and other clusters in the ZS-SEQ-HCC cohort. The significantly different genes between cluster 2 and other clusters are shown with asterisk. Student’s test. (G) Schematic pathway showing key genes in tryptophan metabolism. (H) Boxplots show relative mRNA expression of key genes of tryptophan metabolism in cluster 2 and other clusters in the ZS-SEQ-HCC cohort. Student’s test. (Level of significance: ***P < 0.001; **P < 0.01; *P < 0.05.)

To gain a comprehensive insight into the dysregulation of cellular metabolism in HCCs, we performed untargeted metabolomic profiling and obtained in total 5,890 annotated metabolites in the ZS-SEQ-HCC cohort. The differential analysis and pathway enrichment analysis revealed that the differential metabolites were enriched in glucose metabolism, lipid metabolism, and amino acid metabolism, such as glycolysis and tricarboxylic acid cycle (Fig. 4A). We then compared the metabolite abundances across clusters. Consistent with increased lipogenic gene expression, various lipids were enriched in cluster 3, including adipic acid, arachidic acid, arachidonic acid, and hexadecanedioic acid. The significant accumulation of metabolites in carbohydrate metabolism was observed in cluster 1 and cluster 4, such as lactate and succinate, whereas cluster 2 was characterized by lower levels of metabolites, especially amino acids (Fig. 4B to D). Subsequently, we found that the differential metabolite abundances between cluster 2 and other clusters were highly enriched in the tryptophan metabolism pathway (Fig. 4E), consistent with the lower level of tryptophan in cluster 2. In the TCGA-HCC cohort, the differential genes between cluster 2 and other clusters were also enriched in tryptophan metabolism (Fig. S6A). Besides, cluster 2 had distinctive expressions of key genes involved in tryptophan metabolism, including IDO1, ALDH2, and DDC, from other clusters in the ZS-SEQ-HCC cohort and the TCGA-HCC cohort (Fig. 4F to H and Fig. S6B and C). All findings above explained the reason why there was a significant metabolic discrepancy between cluster 2 and other clusters, especially in tryptophan metabolism.

### Immune characteristics of metabolic clusters in HCCs

**Fig. 5.**
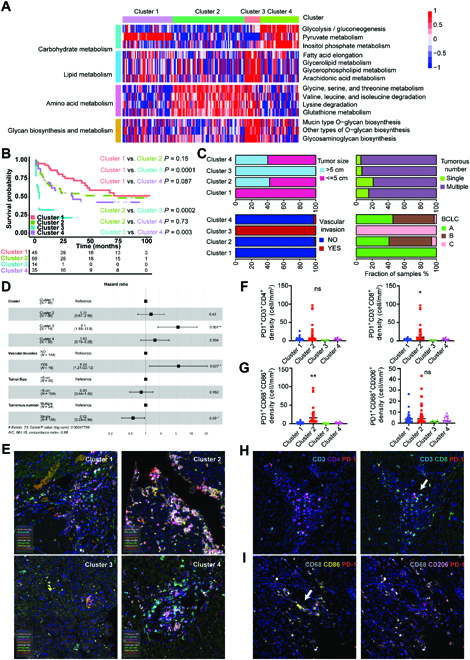
Immune characteristics of metabolic clusters in HCCs. (A) The clustering results based on the enrichment scores of 227 DNBs of HCC tumors in the TCGA-HCC cohort. Heatmap shows normalized enrichment scores of the 4 metabolic clusters in the ZS-HCC cohort. (B) Kaplan–Meier curves of survival among the 4 metabolic clusters in the ZS-HCC cohort. Log-rank test and BH method for adjusting *P* value. Among all clusters, *P* < 0.01; cluster 1 versus cluster 2, *P* = 0.15; cluster 1 versus cluster 3, *P* = 0.0001; cluster 1 versus cluster 4, *P* = 0.087; cluster 2 versus cluster 3, *P* = 0.0002; cluster 2 versus cluster 4, *P* = 0.73; cluster 3 versus cluster 4, *P* = 0.003. (C) Bar plots show the distribution of HCC clinical characteristics among the 4 clusters in the ZS-HCC cohort. (D) Forest plot of multivariate Cox regression analysis for survival adjusting for condition of vascular invasion, tumor size, tumor numbers, and metabolic clusters in the ZS-HCC cohort. The hazard ratios are shown with 95% confidence intervals. *P < 0.05; **P < 0.01; ns, *P* > 0.05. (E) Representative multispectral images of 8 markers on HCC tumor tissues of the 4 clusters in the ZS-HCC cohort. 4′,6-diamidino-2-phenylindole: cyan; CD3: blue; CD4: purple; CD8: green; CD68: white, CD86: yellow; CD20: pink; CD56: orange; and PD-1: red. (F and G) The number or fraction of PD-1^+^ CD4^+^ T cells (CD3/CD4/PD-1) and cytotoxic T cells (CD3/CD8/PD-1)(F) or PD-1^+^ M1 macrophages (CD68/CD86/PD-1) and M2 macrophages (CD68/CD206/PD-1) (G) to investigate which type of immune cells is the main resource for PD-1. (H and I) Representative images of immune cells in cluster 2, (H) CD4^+^ T cells (CD3/CD4/PD-1) and cytotoxic T cells (CD3/CD8/PD-1); (I) M1 macrophages (CD68/CD86/PD-1) and M2 macrophages (CD68/CD206/PD-1).

Recently, it has been proved that the trigger of tryptophan-kynurenine-aryl hydrocarbon receptor pathway could lead to programmed cell death protein 1 (PD-1) upregulation in CD8^+^ T cells and damage their killing effects on tumors in the tumor microenvironment [15,16]. First, 159 HCC tumor samples from ZS-HCC cohort were classified into 4 similar clusters based on using the NSC method through analysis of previous HCC clusters [17] (Fig. 5A and Table S5). We found that cluster 1 and cluster 2 had better survival than the other 2 clusters, while cluster 3 had significantly worst survival (Fig. 5B), consistent with results in the TCGA-HCC cohort (Fig. 2C). Moreover, these clusters had distinct clinical features. Cluster 1 had a higher rate of early-stage patients, smaller tumor size, and less vascular invasion, whereas cluster 3 had a higher rate of terminal-stage patients, bigger tumor size, and more vascular invasion (Fig. 5C). Also, multivariate Cox proportional hazard models revealed that cluster 3 independently predicted a worse prognostic in HCCs (Fig. 5D). Then, we characterized immune cell distribution of these tumor samples and paired normal samples by multiplex immunofluorescence staining, which allowed simultaneous visualization of markers in each formalin-fixed and paraffin-embedded tissue section. In addition to the shape of the nucleus, the marker CD3^+^ was used to identify all T cells, with CD56^+^ as natural killer cell marker and CD68^+^ as macrophage marker. In detail, we defined CD3^+^CD8^+^CD4^−^ cells as cytotoxic T cells, CD3^+^CD8^−^CD4^+^ cells as CD4^+^ T cells, CD68^+^CD86^+^ cells as M1 macrophages, and CD68^+^CD206^+^ cells as M2 macrophages. Representative images of different immune cells with colocalization makers in tumor tissues and normal tissues are shown in Fig. 5E and Fig. S8A, respectively. We found that among these clusters, cluster 2 had an enrichment of immune cells in tumors and normal tissues, though without significant differences (Figs. S7A to C and S8B to D). Interestingly, the density of PD-1-expressing immune cells was higher in cluster 2 than in other clusters (Figs. S7D and S8E). We then found that the density of PD-1^+^ immune cells was highly associated with densities of cytotoxic T cells and M1 macrophages in tumor tissues (Fig. 5F and G and Fig. S7E). Representative images with colocalization makers in tumor tissues are shown in Fig. 5H and I and Fig. S7F. The dysregulated tryptophan metabolism might be an underlying mechanism of the rising density of PD-1^+^ immune cells in cluster 2. In addition, we examined the distribution of other immune checkpoints in immune cells, including programmed cell death ligand 1 (PD-L1), lymphocyte activating gene 3 (LAG-3), and fibrin original protein 1 (FGL1), which attracted us before. We found the density of PD-L1-expressing tumor cells was higher in cluster 2, while no significant difference among clusters was found in the other 2 checkpoints (Fig. S9A to D). Hence, we assumed that HCC patients of cluster 2 might benefit more from PD-1 treatment than other clusters.

### Metabolic clusters have distinct sensitivity to various drugs

Tumor formation and growth involve in various biological metabolism processes. A pool of drugs has been developed to target tumor energy metabolism. We first analyzed transcriptomic and metabolomic data of 12 human HCC cell lines from the Cancer Cell Line Encyclopedia (CCLE). Through the NSC method, HCC cell lines were classified into 4 clusters (Table S6), with Huh7 in cluster 1, PLC/PRF/5 in cluster 2, SKHEP1 in cluster 3, and Hep3B in cluster 4 (Fig. S10A). Consistent with clusters in HCC tissues, the clusters of HCC cell lines also had distinct transcriptional and metabolic profiles (Fig. S10A and B). Notably, the abundances of metabolites in amino acid metabolism were significantly different between cluster 2 and other clusters (Fig. S10B).

**Fig. 6.**
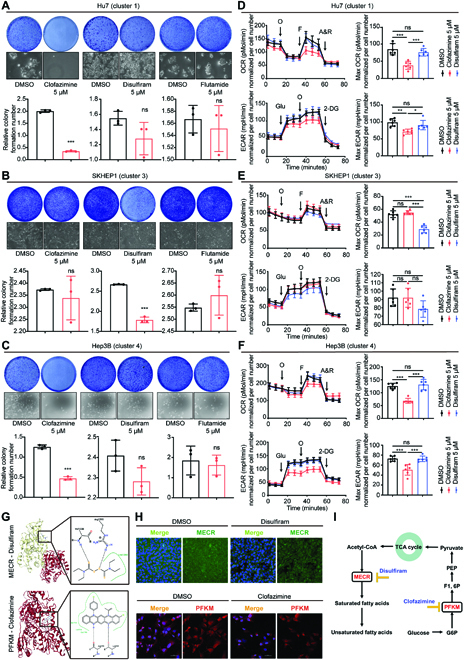
Metabolic clusters have distinct sensitivity to various drugs. (A to C) Representative pictures of colony formation assays and quantification of colony formation assays with different drug treatments in Hu7 (A), SKHEP1 (B), and Hep3B (C). Data are presented as the means ± SD of 3 independent experiments; Student’s test. (D to F) Oxygen consumption rate (OCR) and extracellular acidification rate (ECAR) levels were measured using the Seahorse assay, and the basal/maximal respiration and glycolysis levels were calculated accordingly with different drug treatments in Hu7 (D), SKHEP1 (E), and Hep3B (F). Data are shown as the means ± SD of 6 independent experiments; 1-way analysis of variance and Tukey’s post hoc test. (G) Molecular docking simulation between protein and drug. Upper, MECR and disulfiram; lower, PFKM and clofazimine. (H) Changes in MECR or PFKM with disulfiram or clofazimine treatment were detected by immunofluorescence assay. (I) Schematic pathway showing the targets of clofazimine and disulfiram in metabolism. (Level of significance: ***P < 0.001; **P < 0.01; *P < 0.05; ns, *P* > 0.05.)

We then investigated the drug sensitivity among clusters of HCC cell lines. We selected clofazimine, disulfiram, flutamide, and all-trans retinoic acid (ATRA) for further analysis, which are known anticancer drugs and could target cancer energy metabolism in different ways. According to the drug sensitivity data (Fig. S10C), we found that clofazimine was more effective in cluster 1 and cluster 4, with flutamide and ATRA effective in cluster 4 and disulfiram effective in cluster 3. Notably, our previous study has identified that ATRA could not impact the proliferation and metastasis of HCC cell lines [18]. Therefore, we used clofazimine, disulfiram, and flutamide for further validation. In colony formation assays and Seahorse experiments, we observed the inhibitory effects of clofazimine on the growth, respiration, and glycolysis levels of cell lines within cluster 1 and cluster 4, as well as the efficacy of disulfiram on the growth and respiration levels of cell lines within cluster 3, whereas flutamide could not function in any HCC cell line (Fig. 6A to F and Fig. S11A and B). By predicting the docking between drugs and protein involved in glycolysis and lipid metabolism, we found that disulfiram and clofazimine had a highest binding efficacy with mitochondrial trans-2-enoyl-CoA reductase (MECR) and 6-phosphofructokinase, muscle type (PFKM), respectively (Fig. 6G). Immunofluorescence assay confirmed our docking prediction that clofazimine and disulfiram acted on PFKM and MECR separately (Fig. 6H and I).

Interestingly, we found that compared with other clusters, many stemness-associated genes were more dysregulated in cluster 3 of the ZS-SEQ-HCC cohort, including ARPC5L [19], ASAP1 [20,21], CLDN4 [22], ELF3 [23,24], MAP2K2 [25], and PRKCD [26] (Fig. S12A and B). The abnormal stemness gene expressions may play a part in the worst survival outcome of cluster 3. Besides, the role of stemness genes in causing sorafenib resistance by dysregulating lipid metabolism and oxidative phosphorylation has been reported [27,28]. Subsequently, we used 3-dimensional fibrin gels to sort tumor-repopulating cells from SKHEP1 cells and found that disulfiram enhanced the sensitivity of HCC cells to sorafenib and reduced drug resistance (Fig. S12C to E). In conclusion, these results provide us with a great reference for precision treatments based on our metabolic-pathway-based clusters.

### Potential of metabolic clusters in precision therapy in vivo

**Fig. 7.**
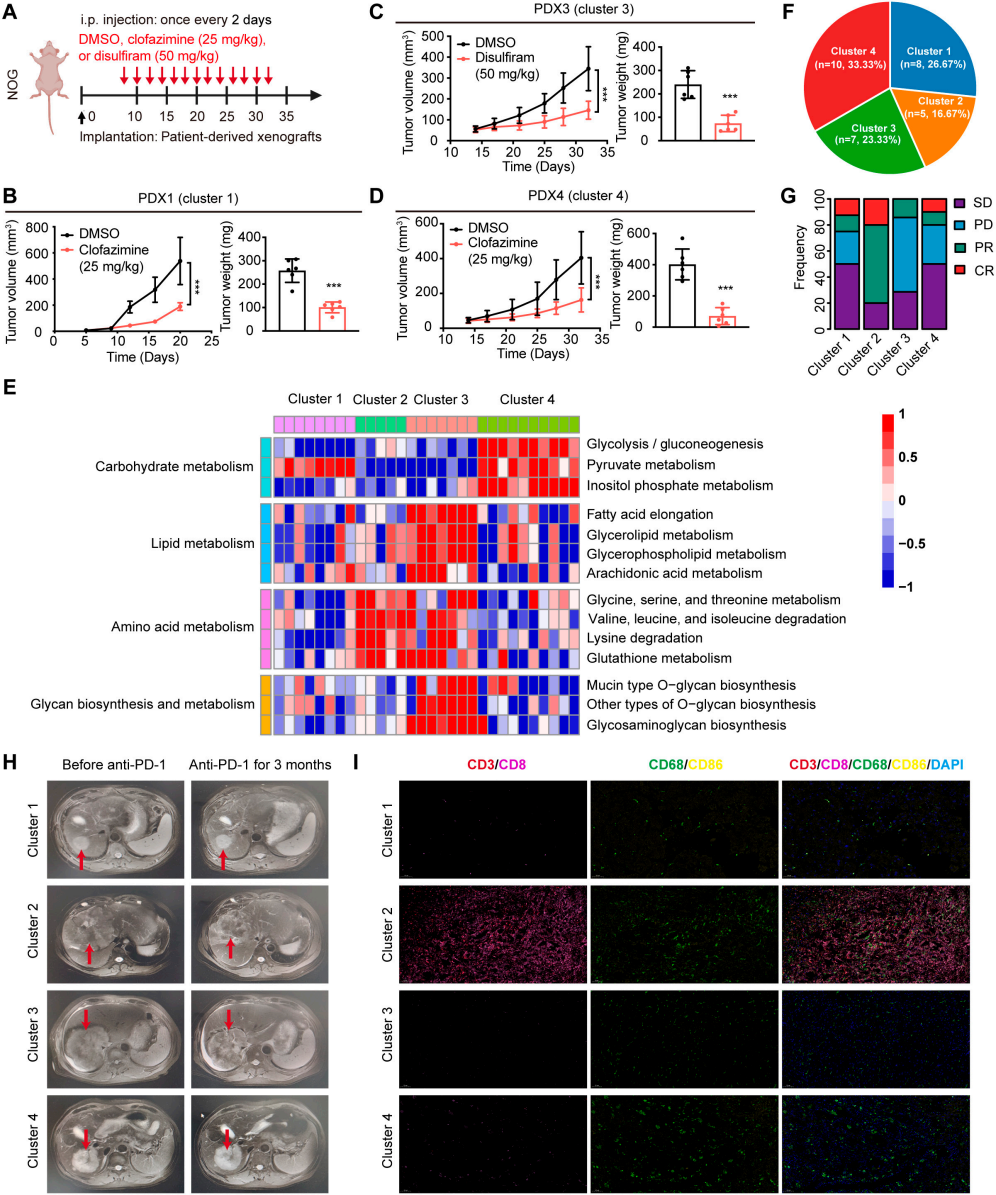
Potential of metabolic clusters in precision therapy. (A to D) Scheme representing the experimental procedure (A), tumor volume and tumor weight of patient-derived xenograft (PDX) models established by implantation of PDXs into NOG mice injected with dimethyl sulfoxide (DMSO), clofazimine (25 mg/kg), or disulfiram (50 mg/kg) (*n* = 6). Cluster 1 / PDX1 (B), cluster 3 / PDX3 (C), and cluster 4 / PDX4 (D). Student’s test. ***P < 0.001. Data are presented as the means ± SD. (E) The clustering results based on the enrichment scores of HCC tumors in the TCGA-HCC cohort by using the NSC method. Heatmap shows normalized enrichment scores of the 4 clusters in the ZS-PD1-HCC cohort (PD-1, 200 mg, every 3 weeks). (F) Pie chart shows the proportions of HCC tumors in each cluster. (G) Bar plots show the effect of PD-1 treatment among the 4 clusters. Chi-square test. *P < 0.05. (H) Representative imaging pictures showing the effect of PD-1 treatment in each cluster. The arrows point to the location of the tumors. (I) Changes of immune cells in the tumor microenvironment after PD-1 treatment are detected by multiplex immunofluorescence assay.

We further validate potential of HCC clusters in precision therapy in vivo by using the previously established HCC patient-derived xenograft (PDX) models [18]. PDX1 were established using the xenografts from patients classified to cluster 1, PDX3 were established using the xenografts from patients classified to cluster 3, and PDX4 were established using the xenografts from patients classified to cluster 4. These PDX models derived from these patients of the ZS-SEQ-HCC cohort. Then, PDX1 and PDX4 were treated with clofazimine, while PDX3 were treated with disulfiram. We found that clofazimine inhibited tumor growth in cluster 1 and cluster 4, and disulfiram inhibited the tumor growth in cluster 3 (Fig. 7A to D), consistent with those results in vitro.

Considering that cluster 2 had more PD-1^+^ immune cells and infiltration, we hypothesized that cluster 2 would be more sensitive to PD-1 therapy. By using the NSC method, 30 HCC samples from ZS-PD1-HCC cohort were classified into 4 clusters. These samples also had unique transcriptional profiles in metabolism (Fig. 7E). Among them, 16.67% of patients was classified to cluster 2 (Fig. 7F). According to the clinical outcomes, we found that patients in cluster 2 got more partial remission or complete remission effects (Fig. 7G), indicating that PD-1 treatment was more effective in cluster 2. Imaging data also suggested that patients in cluster 2 had greater tumor necrosis and shrinkage after PD-1 treatment (Fig. 7H), and multiplex immunofluorescence staining showed more immune cell infiltrations in cluster 2 after treatment, including cytotoxic T cells (CD3^+^CD8^+^) and M1 macrophages cells (CD68^+^CD86^+^) (Fig. 7I). Together, our results indicated the potential of metabolic clusters as an auxiliary means for precision therapy.

## Discussion

Accumulating reports have shown that metabolic reprogramming is associated with disease progression, clinical outcomes, and treatment responses in various cancers [29]. However, the dynamics and heterogeneity of tumor metabolism are important for early diagnosis and prevention of HCC. In this study, there was no significant difference in metabolic gene expression among different stages of HCC when we used the traditional differential gene analysis from TCGA cohort at first. Therefore, we introduced the concept of blast crisis based on DNBs and established a prediction model based on DNBs. Compared with traditional molecular biomarkers, DNBs showed its superiority in identifying critical states in disease progression in a dynamic manner. To our knowledge, this is the first systematic analysis to show the extent of metabolic reprogramming and heterogeneity based on the DNBs method in HCC. However, several published metabolic subtypes were established on the basis of the characteristics of HCC gene or protein expression, which ignore the driver genes of HCC metabolism or do not reflect these gene regulated immune microenvironment well [17,30]. With the DNB method, we identified in total 227 substantial metabolic genes and successfully classified HCC into 4 heterogeneous clusters through these metabolic genes with different metabolic characteristics, prognoses, genomic changes, immune microenvironment, and sensitivity to metabolic inhibitors and immunotherapy.

Consistent with previous studies, we demonstrated that HCC exhibited dysregulations of lipid, amino acid, and carbohydrate metabolisms [31–33]. We then found the dynamic changes of metabolic genes during different HCC stages and identified in total 227 DNBs for predicting tipping point. According to the enrichment scores of metabolic pathways assigned by DNBs, we grouped HCC samples into 4 clusters that had distinct metabolic characteristics. Notably, cluster 3 with the mixed metabolic dysregulation had worst outcomes. These results indicate that metabolic dysregulation play an important role in HCC prognosis. Moreover, the association between clusters and clinical characteristics of HCC might be helpful to understand the metabolic heterogeneity of HCC better and provide biological significance to HCC treatment (Table).

**Table.** Summary of clinical, metabolic, and genomic characteristics and potential therapeutic strategies of the metabolic pathway subtypes of HCC.

Subtype	Cluster 1	Cluster 2	Cluster 3	Cluster 4
Clinical	Good prognosis (5-year RFS, 64%); more BCLC A	Good prognosis (5-year RFS, 58%); multiple tumor.numbers	Poor prognosis (5-year RFS, 29%); bigger tumor size; vascular invasion	Good prognosis (5-year RFS, 47%)
Genomic	Frequent CNV gain in TP53 pathway	Frequent CNV and SNV	Dysregulation of cancer stem genes	N/A
Metabolic Features	Dysregulation of glycolytic genes and intermediate metabolites	Dysregulation of amino acid genes and intermediate metabolites	Dysregulation of lipogenesis genes and intermediate metabolites	Dysregulation of glycolytic genes and intermediate metabolites
Treatment	Clofazimine; glycolysis inhibitors	Anti-PD-1	Disulfiram; lipid synthesis inhibitors	Clofazimine; glycolysis inhibitors

CNV, copy-number variation; SNV, single-nucleotide variants; RFS, relapse-free survival; N/A, not available.

Next, we performed the WES to identify the molecular drivers of different metabolic clusters in HCC. Our results proved that cluster 2 had a relatively higher CNV burden and mutation burden with more frequent mutations in the Hippo and PI3K pathways. Moreover, there were more somatic copy-number alterations in amino acid metabolism genes in cluster 2. Especially, pathway enrichment analysis based on differential metabolites and differential metabolic genes showed that the tryptophan metabolism was the top enriched pathway in cluster 2, with its related metabolic genes dysregulated significantly. The WES analysis implied that genomic alterations in specific chromosomal regions might mediate the metabolic reprogramming and heterogeneity of HCC.

Our multiplex immunofluorescence staining showed that cluster 2 had an enrichment of immune cells in tumor tissues, especially PD-1-expressing cells. The results might be explained by enriched tryptophan metabolism in cluster 2 of the TCGA-HCC cohort and ZS-SEQ-HCC cohort. Recently, many studies have found that enzymes and metabolites of tryptophan metabolism are widely involved in the regulation of immune system [34,35]. IDO1 as the key enzyme in tryptophan metabolism plays a vital role in differentiating CD4^+^ T cells into regulatory T cells in tumors, thereby promoting the immunosuppressive state of the tumor microenvironment [36]. In addition, IDO1 expression in the tumor can induce expression of PD-1 in T cells [37]. Inhibition of GCH1 by 2,4-diamino-6-hydroxypyrimidine could block IDO1 activity, halt tumor growth, and enhance the tumor response to anti-PD-1 immunotherapy [38]. These findings supported the role of dysregulated tryptophan metabolism in inducing PD-1 expression. We further examined the distribution of other immune checkpoints, including PD-L1, LAG-3, and FGL1. We found that there was a higher density of PD-L1^+^ immune cells in tumor tissues of cluster 2, while no differences were observed in LAG-3^+^ and FGL-1^+^ cell densities between cluster 2 and other clusters. Previous studies reported that patients with high expression of PD-L1 and low expression of LAG-3/FGL1 had a better prognosis [39], explaining why cluster 2 had a higher survival. In the future, we will analyze more common immune checkpoints in HCCs to perfect our research results.

We then found that stemness-associated genes [27] were more dysregulated in cluster 3 when compared with other clusters. The abnormal expression of stemness genes can lead to the activation of lipid metabolism pathways and ultimately enhance self-renewal ability and drug resistance of tumor cells [28], explaining why patients in cluster 3 had worst outcomes.

Our study also indicates the application value of identified metabolic-pathway-based clusters in clinical translations, such as therapeutic strategies. The combination of experimental results and in silico modeling demonstrated that the sensitivity of these 4 metabolic clusters to drugs depends on their metabolic features. Clofazimine blocked tumor growth in cluster 1 and cluster 4 through inhibiting glycolytic reprogramming, while disulfiram inhibited tumor growth in cluster 3 by disturbing oxidative phosphorylation. Through simulating the docking between drug molecule and metabolic enzymes and performing experiments, we demonstrated that clofazimine interacted with PFKM [40], a key glycolytic enzyme, and disulfiram interacted with MECR [41], a key lipogenic enzyme. Notably, we used 3-dimensional fibrin gels to sort tumor-repopulating cells from SKHEP1 cells (cluster 3) and found that disulfiram could enhance the antitumor effect of sorafenib and reduce drug resistance, which might be results from inhibitory effect of disulfiram on lipid metabolism in cluster 3. The understanding of dysregulated metabolic networks provided a reference for personalized therapy. At present, the construction of rodent hepatoma model and the research progress of drugs provide a basis for the development of advanced agents with clinical therapeutic potential for liver cancer [42]. In this paper, PDX mouse models were used in order to evaluate potential of metabolic clusters in precision therapy in vivo*.* Considering that the metabolic characteristics in cluster 2 may promote the expression of PD-1, we supposed that HCC patients in cluster 2 would benefit more from PD-1 treatment. Expectedly, in the ZS-PD1-HCC cohort, HCC patients in cluster 2 had a better response to PD-1 treatment, supporting our assumption.

In conclusion, our study revealed the metabolic reprogramming and heterogeneity in HCC, identified 4 clusters by DNB method with distinct metabolic features, genomic alterations, immune microenvironment, and sensitivity to drugs and immunotherapy by DNB method, and suggested the potential of metabolic features as a therapeutic strategy in HCC treatment.

## Materials and Methods

### Sample collection

A total of 219 patients with HCC who underwent curative resection between January 2009 and January 2010 were enrolled in 3 independent cohorts at the Zhongshan Hospital of Fudan University (Shanghai, China), the First Affiliated Hospital of Wenzhou Medical University (Zhejiang, China), and eastern Hepatobiliary Surgery Hospital (Shanghai, China). Fresh tumor liver tissues were collected for analysis at transcriptional or metabolomic level. Ethical approval for the study was obtained from the First Affiliated Hospital of Wenzhou Medical University Ethics Committee. All patients who had not yet received drug treatment were identified by the pathologic diagnosis of HCC. The cohort 1 named as the ZS-SEQ-HCC cohort consists of 30 patients with transcriptomics, metabolomics, and whole-exome capture sequencing analysis. The cohort 2 named as the ZS-HCC cohort is composed of 159 patients with transcriptomics, tissue microarray, and immunohistochemical analysis. The cohort 3 named as the ZS-PD1-HCC cohort consists of 30 patients who accepted PD-1 immunology treatment and transcriptomics analysis (PD-1, 200 mg, every 3 weeks). The cohort 4 named as TCGA-HCC cohort consists of 343 HCC patients with tumor and paired nontumor samples. The transcriptomics sequencing data and clinical data of the TCGA-HCC cohort were collected from the TCGA database (https://portal.gdc.cancer.gov/).

All diagnoses of HCC were based on histopathology and followed World Health Organization criteria. Tumor grades were assigned using the system Edmondson–Steiner system, and Child–Pugh scores were used for liver function assessment. Tumor stage was determined using the Union for International Cancer Control Tumor, Node, Metastasis Classification system. Tumor recurrence was diagnosed on the basis of computed tomography scans, magnetic resonance imaging, digital subtraction angiography, and elevated serum alpha-fetoprotein levels, with or without histological confirmation.

Patients who underwent palliative surgery only, had prior interventions (e.g., trans-hepatic artery embolization, chemotherapy, or radiotherapy), or were diagnosed with other primary malignancies or inflammatory diseases during the follow-up were excluded from the study.

### Differential metabolic genes in TCGA-HCC cohort

On the basis of the clinical data of the TCGA-HCC cohort, HCC samples were divided into 4 groups (Normal, Stage I, Stage II, and Stage III). We performed pairwise comparisons using R package “limma” and selected genes with significant difference (adjusted *P* < 0.05) in at least 1 comparison. *P* values were adjusted for controlling the high false-positive rate in multiple comparisons by using the Benjamini–Hochberg (BH) method. The metabolic pathways and associated genes were downloaded from the KEGG database (www.kegg.jp/). The differential genes that involve in metabolic pathways in the KEGG database were selected. Finally, we obtained 9,193 differential metabolic genes assigned to 344 metabolic pathways for later analysis.

### Calculation of the global divergence between a pair of expression profiles

The global divergence between a pair of gene expression profiles was calculated as Euclidean distance:

RMSD $=\sqrt{\sum_{i=1}^{n}(\log_{2}x_{i}-\log_{2}{y_{i}}^{2}/n}$ , where *x*_i_ and *y*_i_ are the expression of gene *i* in 2 expression profiles, respectively, and *n* is the number of genes present in the expression profile.

### GSEA

The GSEA (“GSEAbase” package in R) was performed on genes preranked by gene-expression-based log2 fold change between tumors and normal tissues. *P* values were adjusted for controlling the high false-positive rate in multiple comparisons by using the BH method. The results with FDR below 0.05 were considered significantly differential pathways. The enriched pathways were classified into 8 metabolic categories according to the KEGG database.

### DIRAC analysis

DIRAC analysis (“GSReg” package in R) was performed to quantify the variability of various biological pathways or network across individuals. The gene expression profiles and metabolic pathways information would be used to calculate RCI of each pathway through gene ranking and pairwise comparison of gene expression. RCI represents the degree of pathway-level perturbations between samples in a given phenotype (group). An RCI of 1.0 indicates mostly unchanged ranks of pathway genes among samples, and an RCI of 0.5 indicates greatly variable ranks of pathway genes among samples. In this study, we used the 344 metabolic pathways consisting of 9,193 metabolic genes to calculate the RCIs in normal samples or HCC samples of the TCGA-HCC cohort.

### Dynamic changes analysis and DNB analysis

We performed dynamic changes analysis (“Mfuzz” package in R) on the metabolic gene expression profiles to get gene clusters and their changes during different stages. The optimal number of clusters was obtained using “Dmin” function in R package “Mfuzz”. We then used the metabolic gene expression profiles to identify DNB according to the nonlinear dynamic theory. The DNBs is a group of molecules or genes that satisfy the following 3 criteria:

• The expressions of DNB members fluctuate widely, represented by the SD.
• Pearson correlation coefficients (PCC*i*) among DNB members at the mRNA expression level are increased significantly.
• Pearson correlation coefficients (PCC*o*) between DNB members and non-DNB genes are decreased significantly.

The CI was calculated as the numerical signal of the DNB method:

$$\text{CI}=\frac{\mathrm{PCC}\text{in}}{\mathrm{PCC}\text{out}}\;\mathrm{SD}i$$

where PCC*in* is average PCC*i* (in absolute value) of all pairwise correlation in DNBs, PCC*out* is average PCC*o* (in absolute value) of all pairwise correlation between DNBs and other genes, and SD*i* is the average SD of DNB members.

We performed the following steps to identify DNB members at each HCC stage (Stage I, Stage II, and Stage III) with normal group as a reference group: (1) calculate SD of each metabolic gene at normal group and each HCC stage, and choose genes whose SD in a given stage are at least 2 times more than SD in normal group; (2) cluster the selected genes in the above step at each HCC stage (“hclust” function in R with method as “complete”); (3) normalize the expression data of each gene at each HCC stage by Fisher z transformation; (4) calculate the 4 indices of each cluster at normal group and each HCC stage: average SD, average absolute Pearson’s correlation coefficient (|PCC|) among the cluster members, average |PCC| between the cluster members and other genes, and CI; (5) select cluster that match 3 criteria of DNB and have a highest CI, and regard the corresponding stage as a candidate stage.

Finally, we obtained 227 genes as DNBs and identified Stage II as a candidate stage. For constructing gene networks, we collected latest information of human protein-protein interaction network from the string database (www.string-db.org/, 2021.10). The visualization of networks was achieved by Cytoscape (version 3.7.1).

### GSVA and metabolic-pathway-based clustering

We used GSVA (“GSVA” package in R) to calculate the enrichment scores of 155 metabolic pathway assigned by 227 DNBs in each sample of the TCGA-HCC cohort. Before clustering, we scaled the enrichment scores profile of each sample. The *k*-means clustering (“kmeans” function in R) was performed to cluster 343 HCC samples based on Euclidean distance of enrichment scores profile, with k in the range of 2 to 8. We then used consensus clustering (“ConsensusClusterPlus” package in R) to assess the robustness of clustering, with iteration as 1,000 and resampling as 80%. We applied 21 different testing methods by using NbClust testing (“NbClust” package in R) for determining the optimal number of clusters. The stability of clustering was confirmed by Silhouette analysis.

### Classification of HCC patients and their HCC cell lines

We firstly removed batch effects of mRNA expression data of HCC patients in other cohorts and HCC cell lines with expression data of HCC samples in the TCGA-HCC cohort as a reference (“ComBat” function in R package “sva”). Then, each mRNA expression dataset was normalized (“scale” function in R). The enrichment score of each metabolic pathway in each sample was calculated via the GSVA method. Then, we trained a model based on enrichment score profiles and clustering information of the TCGA-HCC cohort and utilized the model to classify other HCC patients and HCC cell lines to identified metabolic-pathway-based clusters, by using the NSC method in the R package “pamr”.

### Transcriptome sequencing

After finishing RNA sample isolation, double-stranded complementary DNA (cDNA) was synthesized using an mRNA template after mRNA enrichment by Oligo (dT) magnetic beads. The purified double-stranded cDNA later underwent terminal repair, poly-A tail addition, and adapter incorporation. The cDNA was screened by AMPure XP beads, amplified by polymerase chain reaction, and purified by AMPure XP beads again. Finally, a library was constructed. After checking the quality of the library, the high-throughput sequencing platform was used to sequence the library. The libraries were sequenced on an Illumina HiSeq X Ten platform, and 150 bp paired-end reads were generated. Clean data were obtained for downstream analyses by removing reads containing adapter, reads containing ploy-N, and low-quality reads from raw data using Trimmomatic. The clean reads were mapped to the human genome (hg38p13) using HISAT2. FPKM value of each gene was calculated using Cufflinks, and the read counts of each gene were obtained by HTSeq-count.

### WES and statistical analysis

The WES process includes 2 main modules: library construction and sequencing. Agilent SureSelect HS Target Enrichment System was used to enrich whole-exon regions, and high-throughput sequencing was performed on NovaSeq 6000 sequencer. The library construction and enrichment were conducted using Agilent SureSelect Human All Exon V6 kit. Sample DNA quality evaluation: 1 μg of DNA samples (Qubit quantitative value), agarose gel electrophoresis quality test without degradation or RNA pollution. Qualified DNA was randomly broken into 150- to 300-bp fragments. After terminal repair, poly-A tail addition, and adapter linkage, we constructed a DNA library. After pooling, the library was hybridized with biotin-labeled exon probes in liquid phase, and then the exon sequences were extracted by streptomycin magnetic beads. After quality control, we used NovaSeq 6000 to perform PE (pair-end) 150-bp sequencing. Every library fragment underwent PE 150-bp sequencing, after which a pair of 150-bp sequences can be obtained and called as reads (the basic unit of sequencing data). According to a previous study [14], there are 10 typical oncogenic signaling pathways consisting of 335 genes. For each sample, we calculated the number of SNV and CNV occurring in the genes that involve in these 10 oncogenic pathways. For each metabolic cluster, we calculated the sample proportion with at least 1 SNV or CNV in each of the 10 oncogenic pathways and performed comparisons in every 2 clusters and among clusters. The tumor sample is thought to have CNV or SNV in the given pathway whose genes have at least 1 CNV or SNV.

### Liquid chromatography-tandem mass spectrometry analysis

The raw data was converted to MZXML format through ProteoWizard. Then, we used XCMS program for peak alignment, retention time correction, and extraction of peak area. The structure identification of metabolites was carried out by using the matching method of accurate mass number (<25 ppm) and secondary spectrum. In addition, the self-built database was retrieved. For the data extracted from XCMS, the missing ion peaks > 50% in the group were deleted. We used SIMCA-P v14.1 (Umetrics, Umea, Swede) for pattern recognition. After the data was preprocessed by Pareto scaling, we performed multidimensional statistical analysis, including unsupervised PCA, supervised least squares discriminant analysis and orthogonal least squares discriminant analysis. The differential metabolites in every 2 clusters or between 1 to other 3 clusters were obtained using Student *t* test. We used Omicsbean to perform KEGG pathway enrichment analysis.

### Docking possibilities between proteins and drugs

We assessed the docking possibilities between the target proteins and small drug molecules by using Autodock software (https://autodock.scripps.edu/), with crystal structure of each protein as the receptor and each drug molecule as the docking ligand. The crystal structures of metabolic genes involved in glycosis and lipid metabolism were downloaded from the Protein Data Bank (PDB, http://www.wwpdb.org/), processed by removing water molecules and heteroatoms, and saved for molecule docking. The structures of 2 small-molecule drugs, clofazimine and disulfiram, was obtained from the ChemSpider database (www.chemspider.com). We used Autodock to perform semiflexible docking, in which the receptor is rigid and ligand is flexible. By evaluating the binding free energy between receptor and ligand, we found that clofazimine and disulfiram exhibited the highest binding efficacy with PFKM (PDB_ID: 4OMT) and MECR (PDB_ID: 2VCY), respectively. We used ProteinsPlus (https://proteins.plus/) to visualize the protein–drug docking results, with PoseView used to generate 2-dimensional diagrams of protein–drug interactions.

### PDX mouse models and drug treatment

We isolated fresh tumor tissues from patients of clusters 1, 3, and 4 in ZS-SEQ-HCC cohort in the operating room and dissected them into 1 mm^3^. We anesthetized the NOG mice and subcutaneously implanted the HCC tissues into the right superior flank of the mice. After 2 months, when the diameter of tumors reached 1 cm, the subcutaneous PDX tumors were removed, which later were dissected into 3 pieces about 2 × 2 × 2 mm in size, and retransplanted to flanks of nude mice for about 30 days for growth. The mice were euthanized no more than 5 weeks, or at the time when tumors reached 10 mm in diameter. For the drug treatment groups, the mice received 25 mg of clofazimine or 50 mg of disulfiram per mouse per kilogram every 2 days via tail intravenous injection on the eighth day after transplantation. For the control group, the mice received isovolumetric dimethyl sulfoxide per mouse per kilogram every 2 days via tail intravenous injection on the eighth day after transplantation.

### Quantification and statistical analysis

Comparisons of continuous variables between 2 groups of normally distributed data were made using Student *t* test, with non-normally distributed data tested by Mann–Whitney test. Comparison among multiple groups were made by 1-way analysis of variance and Tukey's post hoc test. Two-sided *P* values less than 0.05 were considered statistically significant (level of significance: *P < 0.05; **P < 0.01; ***P < 0.001; ns, *P* > 0.05). All results are shown as means ± SD unless otherwise indicated. Survival curves were constructed using the Kaplan–Meier method. The significant differences between 2 or more survival curves were tested using log-rank test. The BH method was utilized in multiple comparisons to decrease false-positive rates. The association between the clinical information and metabolic clusters was examined using the chi-square test. Univariate and multivariate Cox proportional hazard regression models adjusted or not adjusted for available prognostic clinical covariates were performed to calculate hazard ratios and 95% confidence intervals. Correlation analysis was conducted with Spearman’s correlation. All statistical analyses were performed with R software (version 3.6.3) for statistical computing, Python Programming Language (version 3.8), or GraphPad Prism software (version 8.0).

### Other materials and methods

For further details regarding the materials used, please refer to the Supplementary Materials.

**Ethical approval**

The animal study protocols were approved by the Animal Care and Use Committee of Zhongshan Hospital, Fudan University, Shanghai, China. Each patient provided informed consent before they participated in the study. The study protocol was approved by the Ethics Committee of The First Affiliated Hospital of Wenzhou Medical University (Zhejiang, China, No. 123/2020).

## Data Availability

The transcriptomics data and metabolomics data of the ZS-SEQ-HCC cohort as well as the transcriptomics data of the ZS-PD1-HCC cohort have been uploaded to the National Center for Biotechnology Information Gene Expression Omnibus (https://www.ncbi.nlm.nih.gov/geo/), with an accession number of GSE195952. The whole-exome capture sequencing data of the ZS-SEQ-HCC cohort have been uploaded to the Sequence Read Archive database of National Center for Biotechnology Information (https://www.ncbi.nlm.nih.gov/sra), with an accession number of PRJNA802641. The transcriptomics data and clinical and survival data of TCGA-HCC cohort were downloaded from TCGA and University of California Santa Cruz (UCSC) xena (https://xenabrowser.net/datapages/). The scores for tumor purity, presence of stromal cells, and immune cell infiltration in tumor tissues of the TCGA-HCC cohort were obtained from ESTIMATE (https://bioinformatics.mdanderson.org/estimate/). The transcriptomics data, metabolomics data, and drug sensitivity profiling of 12 human HCC cell lines are available in CCLE (https://portals.broadinstitute.org/ccle). All data in the main text and supplementary materials are available upon request.

## Supplementary Materials

Supplementary 1 Fig. S1. The distribution of 3 criteria of identified DNBs in each stage in the TCGA-HCC cohort. Fig. S2. Estimation of the optimal clustering numbers for HCCs in the TCGA-HCC cohort. Fig. S3. Metabolic clusters and mutation profile in the ZS-SEQ-HCC cohort. Fig. S4. Correlation between CNV number and mRNA expression of metabolism-related genes in the ZS-SEQ-HCC cohort. Fig. S5. Genomic alterations of metabolic clusters in the TCGA-HCC cohort. Fig. S6. Detailed metabolic characteristics of cluster 2 in the TCGA-HCC cohort. Fig. S7. Related to Fig. 5. Fig. S8. Immune characteristics of metabolic clusters in tumor-adjacent tissues in the ZS-HCC cohort. Fig. S9. Characteristics of 3 immune checkpoints (FGL1, LAG-3, and PD-L1) of metabolic clusters in the ZS-HCC cohort. Fig. S10. Metabolic clusters of 12 human HCC cell lines from the CCLE. Fig. S11. Effects of metabolic drugs in PLC/PRF/5. Fig. S12. Characteristics of stemness associated genes among metabolic clusters in the ZS-SEQ-HCC cohort.
