# Supplementary material for: Comprehensive Metabolic Profiling and Genome-wide Analysis Reveal Therapeutic Modalities for Hepatocellular Carcinoma
Source: Research (Wash D C). 2023 Jan 13;6:0036. doi: 10.34133/research.0036 (PMC10076022; doi:10.34133/research.0036)
Supplement: Supplementary 1 — Fig. S1. The distribution of 3 criteria of identified DNBs in each stage in the TCGA-HCC cohort. Fig. S2. Estimation of the optimal clustering numbers for HCCs in the TCGA-HCC cohort. Fig. S3. Metabolic clusters and mutation profile in the ZS-SEQ-HCC cohort. Fig. S4. Correlation between CNV number and mRNA expression of metabolism-related genes in the ZS-SEQ-HCC cohort. Fig. S5. Genomic alterations of metabolic clusters in the TCGA-HCC cohort. Fig. S6. Detailed metabolic characteristics of cluster 2 in the TCGA-HCC cohort. Fig. S7. Related to Fig. 5. Fig. S8. Immune characteristics of metabolic clusters in tumor-adjacent tissues in the ZS-HCC cohort. Fig. S9. Characteristics of 3 immune checkpoints (FGL1, LAG-3, and PD-L1) of metabolic clusters in the ZS-HCC cohort. Fig. S10. Metabolic clusters of 12 human HCC cell lines from the CCLE. Fig. S11. Effects of metabolic drugs in PLC/PRF/5. Fig. S12. Characteristics of stemness associated genes among metabolic clusters in the ZS-SEQ-HCC cohort. [file research.0036.f1.docx]

**Supplementary information for the manuscript**

**Comprehensive metabolic profiling and genome-wide analysis reveal therapeutic modalities for hepatocellular carcinoma**

Feng Qi, Jia Li, Zhuoran Qi, Jian Zhang, Bin Zhou, Biwei Yang, Wenxing Qin, Wenguo Cui, Jinglin Xia

Correspondence: Jinglin Xia, xiajinglin@fudan.edu.cn; Wenguo Cui, wgcui80@hotmail.com; Wenxing Qin, qinwenxingqwx@163.com

**Supplementary Methods**

**Multiplex immunofluorescence staining**

We performed antibodies specific CD3 (Rabbit monoclonal, clone SP7, Abcam, Cat# ab16669), CD4 (Rabbit monoclonal, clone EPR19514, Abcam, Cat# ab183685), CD8 (Rabbit monoclonal, clone EPR21769, Abcam, Cat# ab217344), CD56 (Rabbit monoclonal, clone E7X9M, Cell Signaling Technology, Cat# 99746), CD68 (Rabbit monoclonal, clone EPR23917-164, Abcam, Cat# ab283654), CD86 (Rabbit monoclonal, clone EPR21962, Abcam, Cat# ab239075), CD206 (Rabbit monoclonal, clone E6T5J, Cell Signaling Technology, Cat# 24595), PD1 (Rabbit monoclonal, clone E1L3N, Cell Signaling Technology, Cat# 13684) combined immunofluorescence (mIF). The sections were deparaffinated with xylene and then rehydrated with ethanol. We used Tris-EDTA buffer (pH 9.0) and incubated at boiling point for 15 min for antigen repair, and 3% hydrogen peroxide at room temperature for 15 min to block the activity of endogenous peroxidase. We used goat serum solution to block non-specific antigen for 30 min. These sections were incubated overnight at 4 ℃ with primary antibodies, and then added to the horseradish peroxide (HRP)-conjugated secondary antibody at room temperature for 30 min. Next, we incubated sections with Opal tyramide signal amplification (TSA) Fuorochromes (Opal 7-Color Manual IHC Kit, Perkin Elmer, NEL811001KT) at 37 °C for 20 min. Between each run, Ab-TSA complex in sections were removed by microwave and sections were blocked with the goat serum solution. On the last run, 4′,6-diamidino-2-phenylindole, dihydrochloride (DAPI) was added for nuclei visualization and was mounted with glycerin.

**Cell proliferation assays**

Colony formation assays were used to evaluate cell proliferation. 2000 cells/well were planted in a 6-well plate. After 2 weeks of culture, the cells were fixed with Parachrome and stained with Crystal Violet. Photographs were taken and data were collected.

**Oxygen consumption and extracellular acidification.**

Oxygen consumptions rates and extracellular acidification rates were measured using mitochondrial stress tests and glycolysis stress tests, respectively (Seahorse XF24 Cellular Flux Analyzer, Agilent, Santa Clara, Canada). HCC cells were seeded in cell culture microplates (1 × 10^5^ cells per well) and cultured for 24 h. They were then washed using assay medium (non-buffered XF Base Medium Minimum DMEM), cultured at 37 °C for 1 h before assay and added with DMOG (100 μM). During the mitochondrial stress tests, selective inhibitors were injected during measurements to obtain final concentrations of oligomycin (1 μM), FCCP (5 μM), and antimycin (1 μM). Glucose (20 mM), oligomycin (4.5 μM), and 2-DG (100 mM) were injected during the glycolysis stress tests.

**Immunofluorescence (IF)**

Cells were fixed with 4% paraformaldehyde for 20 min at room temperature, washed three times with PBS, and blocked with Immunol Staining Blocking Buffer (P0102; Beyotime) for 1 h at 37 °C. They were then incubated with MECR (Rabbit polyclonal, proteintech, Cat#No.51027-2-AP) or PFKM (Rabbit polyclonal, proteintech, Cat#No.55028-1-AP) antibodies at 4 °C overnight and washed thrice with PBS. The cells were then incubated with a DyLight 549 dye conjugated goat anti-rabbit IgG (1:200; A23320; Abbkine, CA, USA) for 1 h. DAPI (C1002; Beyotime) was used for nuclear staining. Images were captured using the microscope (IX73, Olympus).

**TRCs proliferation**

We treated TRCs which cultured in 3D fibrin gels with agents. 24 hours later, Cell Counting Kit-8 (CCK-8, Dojindo, Japan) was used following manufacturer's protocol to assess the cell viability. And the OD value was measured at 450 nm. The formula for the cell inhibition rate was: [(OD control cells - OD treated cells) / (OD control cells - OD blanks)] × 100. Cell apoptosis assay was assessed by flow cytometry analysis according to the manufacturer’s instructions (Sigma, USA).

**Supplementary Figures**

**
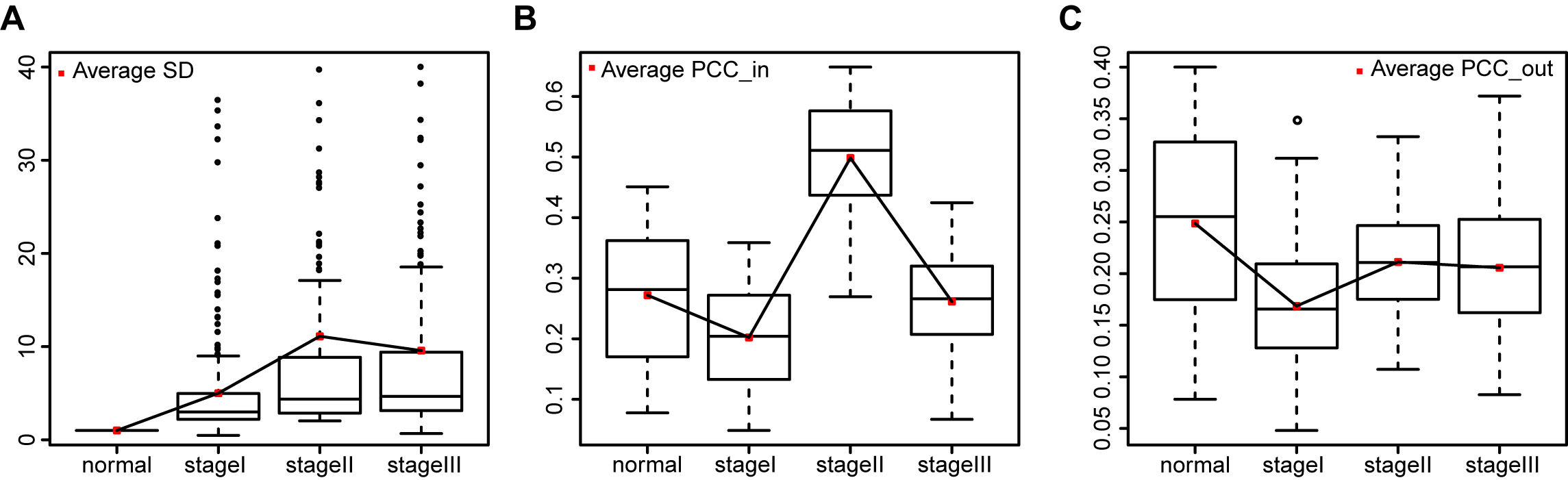
**

**Figure S1. The distribution of three criteria of identified DNBs in each stage in the TCGA-HCC cohort.** (A-C) SD (A), PCC_in (B) and PCC_out (C) of 227 DNBs were calculated within each stage according to three criteria.


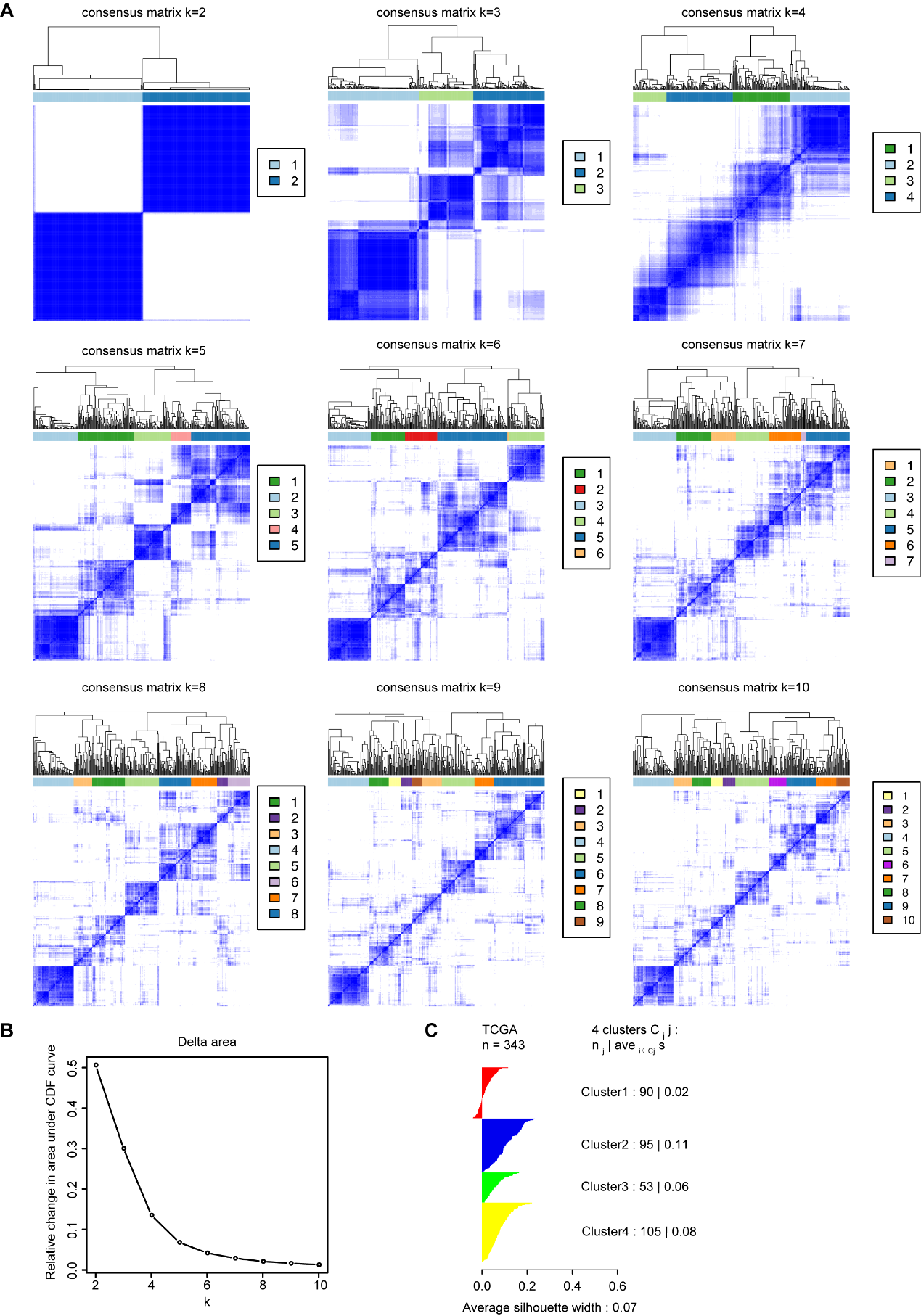


**Figure S2. Estimation of the optimal clustering numbers for HCCs in the TCGA-HCC cohort.** (A) Consensus clustering matrixes based on metabolic-pathway-based enrichment score. The testing range of k is from 2 to 8. (B) Relative change in area under CDF curve. (C) Silhouette analysis of clustering results. The clustering results with k as 4 had best stability.

**
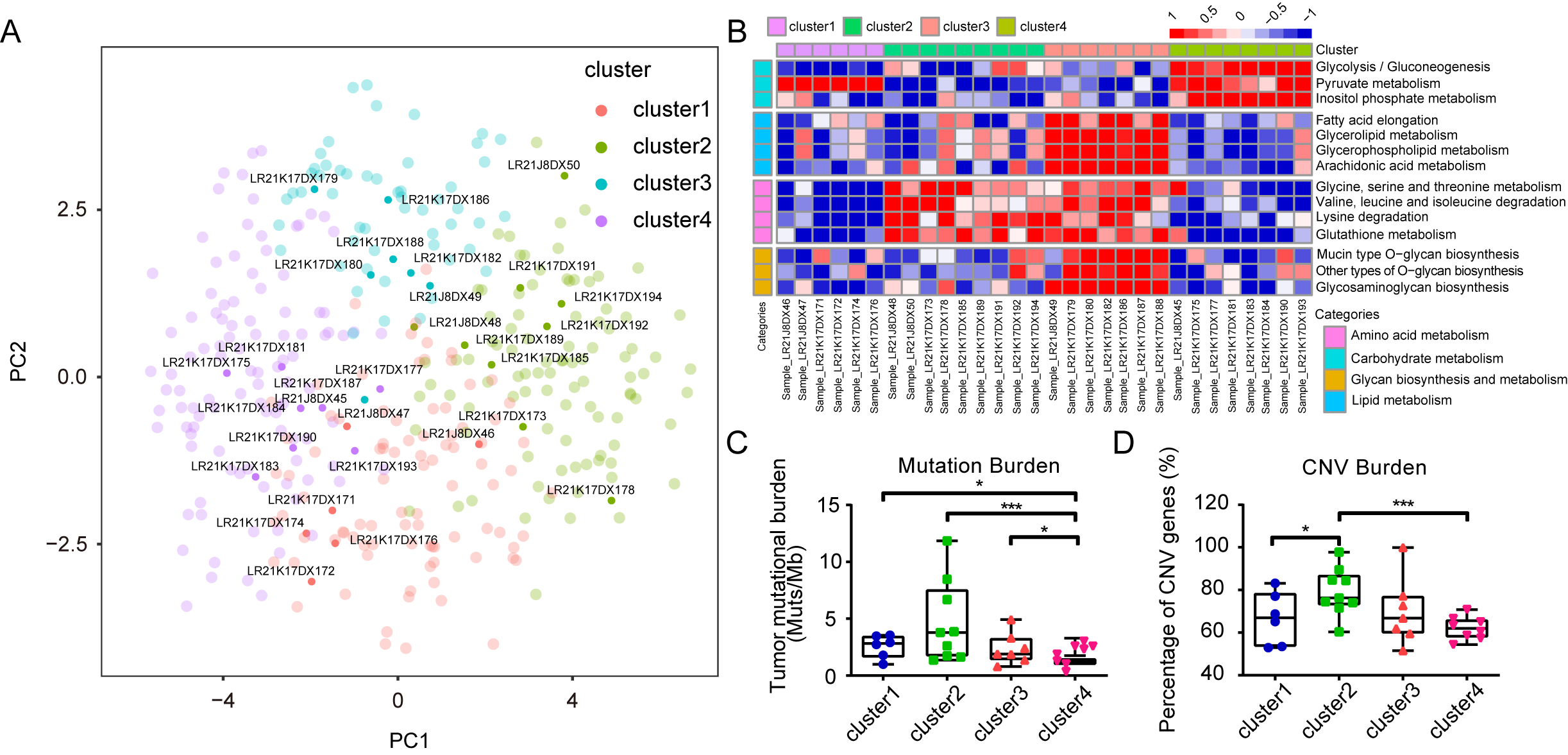
**

**Figure S3. Metabolic clusters and mutation profile in the ZS-SEQ-HCC cohort.** (A) 30 HCC samples in the ZS-SEQ-HCC cohort were classified into four metabolic clusters, based on the nearest shrunken centroids method. (B) Heatmap shows normalized enrichment scores of the four clusters. (C-D) Comparison of CNV burden (C) and mutation burden (D) among the four metabolic clusters. Data are presented as the mean ± SD; Tukey’s post hoc test. ****P* < 0.001; **P* < 0.05.

**
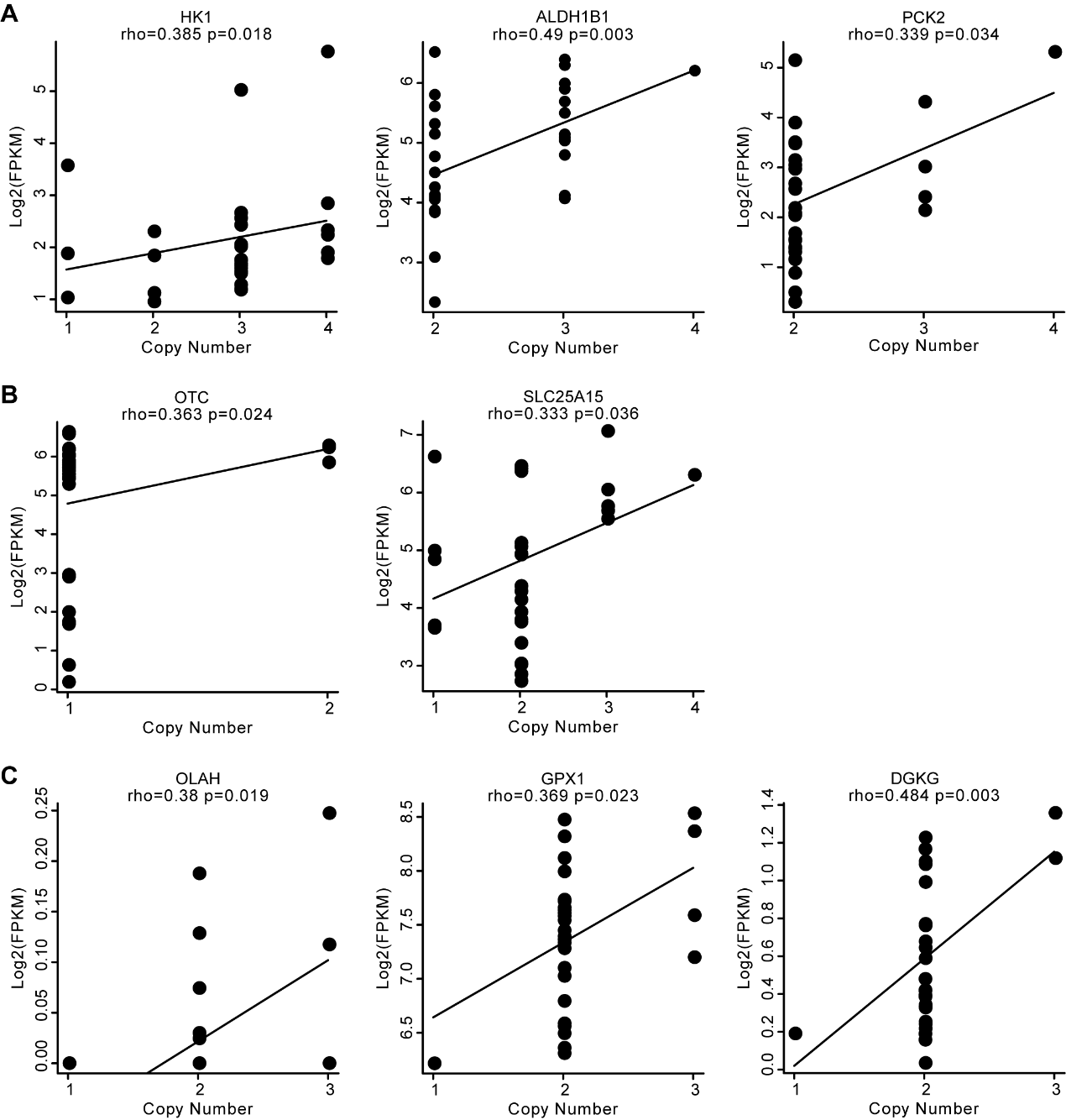
**

**Figure S4. Correlation between CNV number and mRNA expression of metabolism-related genes in the ZS-SEQ-HCC cohort.** (A-C) Scatter plot showing correlation between CNV and mRNA expression of genes involved in carbohydrate (A), lipid (B) and amino acid (C) metabolism. The X-axis represents the copy number. The value below 2 indicates CNV loss. The value over 2 indicates CNV gain. The Y-axis represents the mRNA expression of gene. Each dot represents each sample in the ZS-SEQ-HCC cohort.


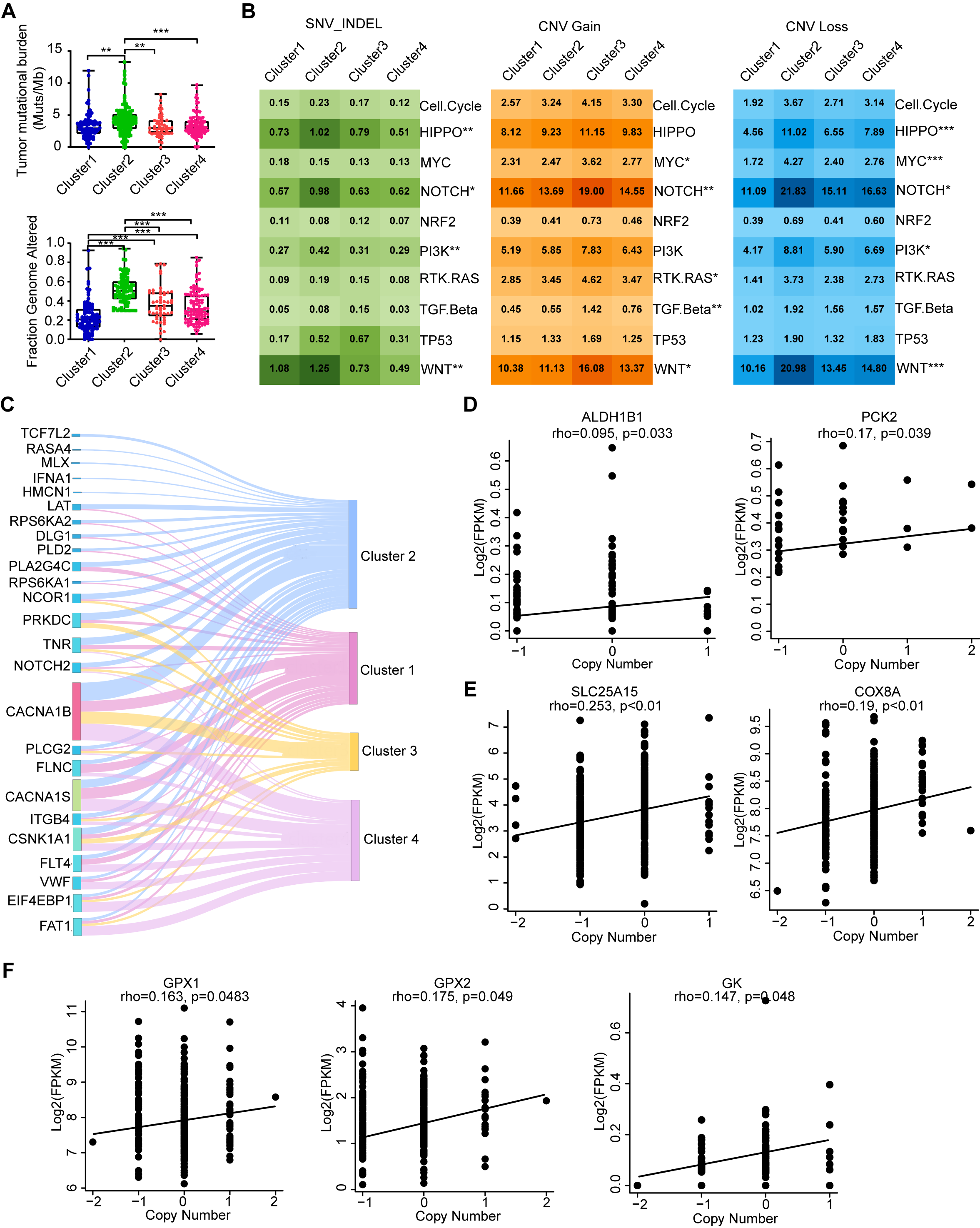


**Figure S5.** **Genomic alterations of** **metabolic clusters in TCGA-HCC cohort.** (A) Comparison of mutation burden (Up panel) and genome altered (Down panel) among the four metabolic clusters. Data are presented as the mean ± SD; Tukey’s post hoc test. ****P* < 0.001; ***P* < 0.01. (B) Genomic alterations in ten oncogenic pathways were compared between all possible pairs of the four metabolic clusters in the TCGA-HCC cohort. The color in the box represents different types of genomic alterations (green, SNV_INDEL; orange, CNV gain; blue, CNV loss), and the color saturation represents the mutation frequency. The pathways with significant differences in mutation frequency in at least one of pairwise comparison among four clusters were labelled with asterisk. (Mann-Whitney test; level of significance: ***, *P* < 0.001; **, *P* < 0.01; *, *P* < 0.05). (C) Sankey diagram for the mutation frequency of genes that showed a significant difference (*P* < 0.05) in the comparison between all possible pairs among the four metabolic clusters in the TCGA-HCC cohort. (D-F) Scatter plot showing correlation between CNV and mRNA expression of genes involved in carbohydrate (D), amino acid (E) and lipid (F) metabolism. The X-axis represents the copy number. The value below 2 indicates CNV loss. The value over 2 indicates CNV gain. The Y-axis represents the mRNA expression of gene. Each dot represents each sample in the TCGA-HCC cohort.


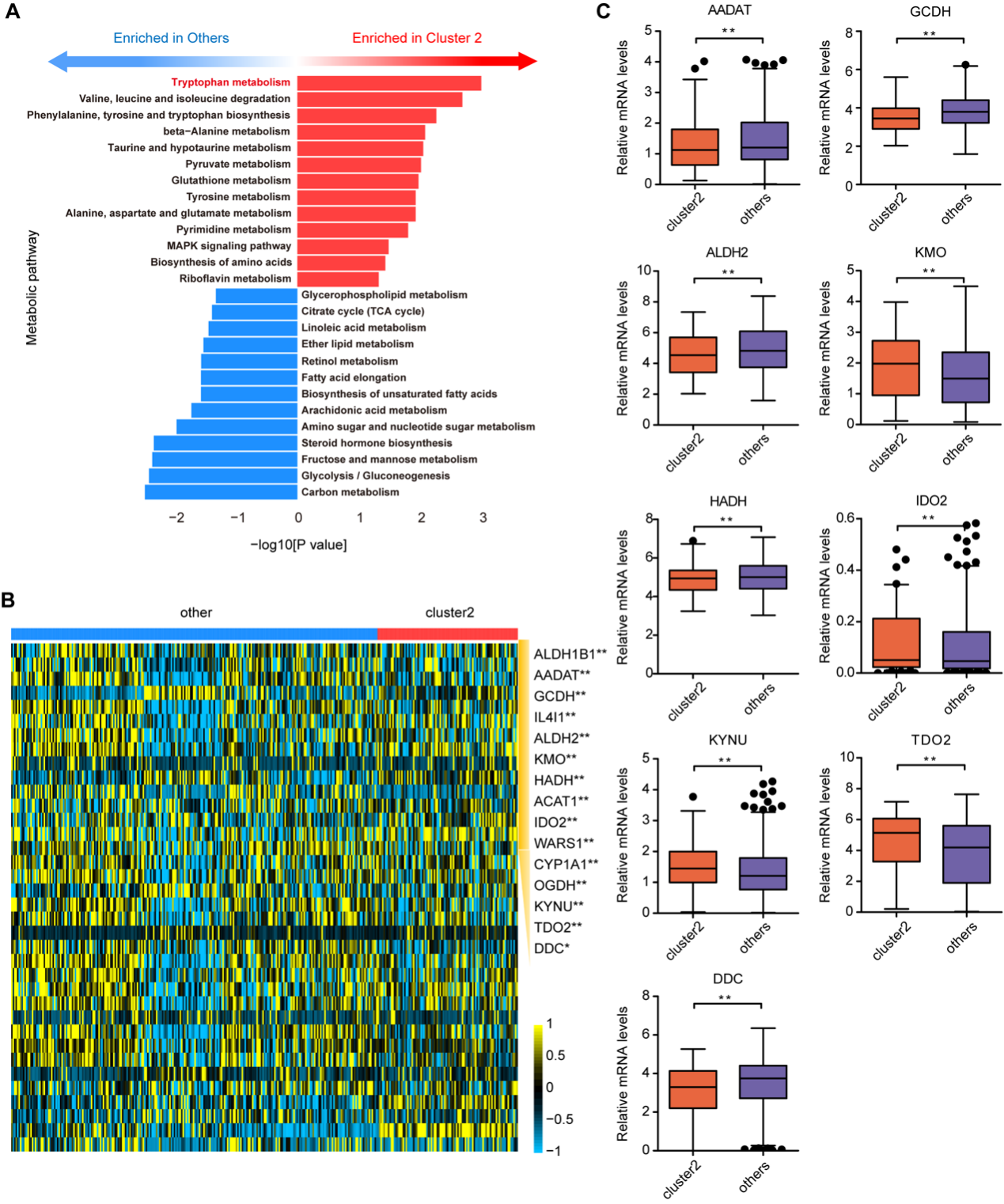


**Figure S6. Detailed metabolic characteristics of cluster 2 in the** **TCGA-HCC cohort.** (A) Barplot shows metabolic pathways enriched by differential metabolic genes between cluster 2 and other clusters in the TCGA-HCC cohort. Red, upregulated pathways in cluster 2; blue, downregulated pathways in cluster 2. (B) Heatmap shows relative mRNA expression of genes related to tryptophan metabolism in cluster 2 and other clusters in the TCGA-HCC cohort. The significantly differential genes between cluster 2 and other clusters are shown with asterisk. Student’s t test. *****P* < 0.0001; ****P* < 0.001; ***P* < 0.01; **P* < 0.05. (C) Boxplots show relative mRNA expression of key genes of tryptophan metabolism in cluster 2 and other clusters in the TCGA-HCC cohort. Student’s t test. ***P* < 0.01.

**
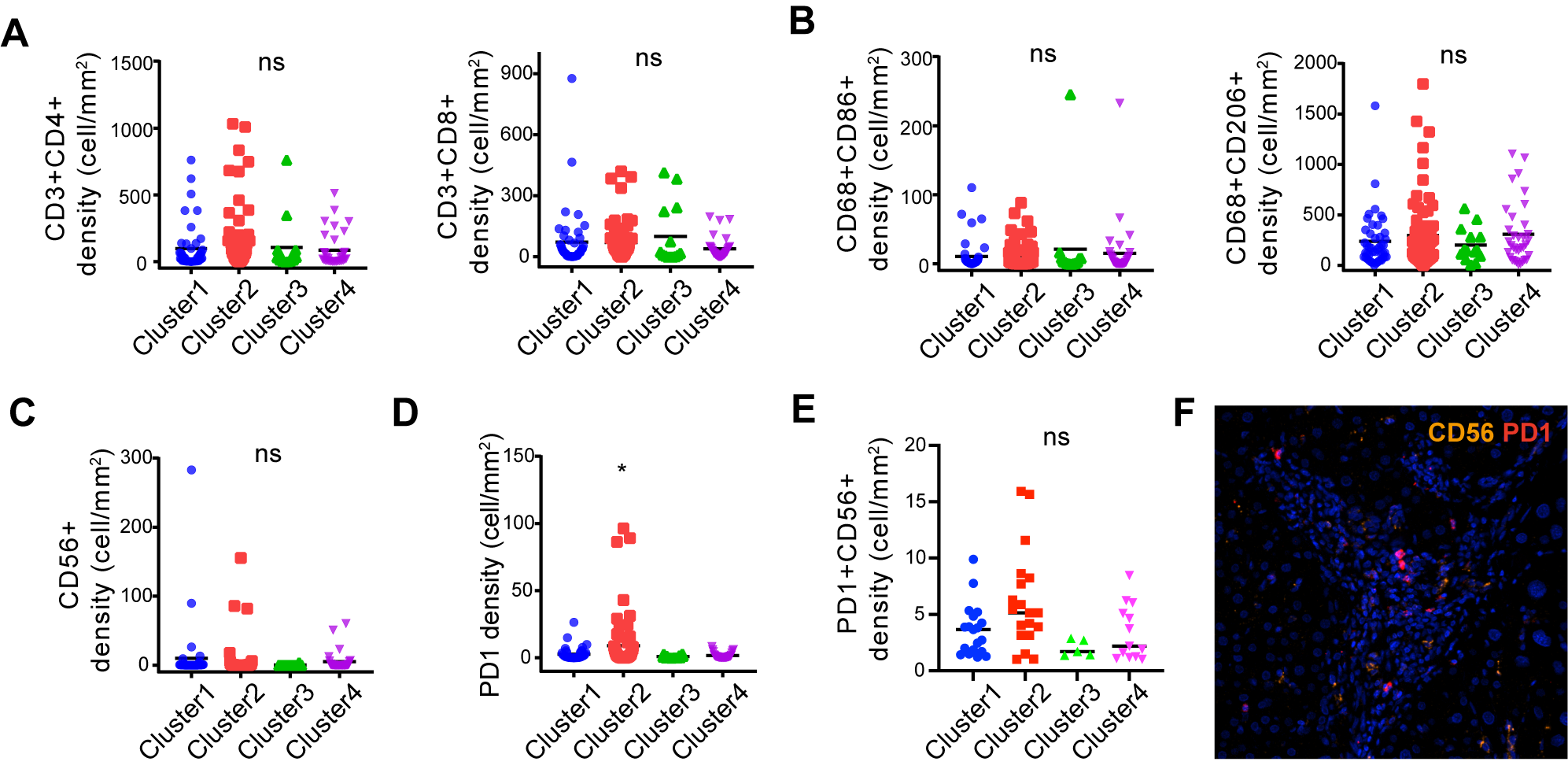
**

**Figure S7. Related to Figure 5.** (A-D) Distribution of different makers among the four clusters in the ZS-HCC cohort. (A) from left to right: CD3+CD4+and CD3+CD8+; (B) from left to right: CD68+CD86+ and CD68+CD206+; (C) CD56+; (D) PD-1. (E) The number or fraction of PD-1+NK cells (CD56/PD-1). Tukey’s post hoc test. **P* < 0.05; ns *P* > 0.05. (F) Representative images of NK cells (CD56/PD-1).

**
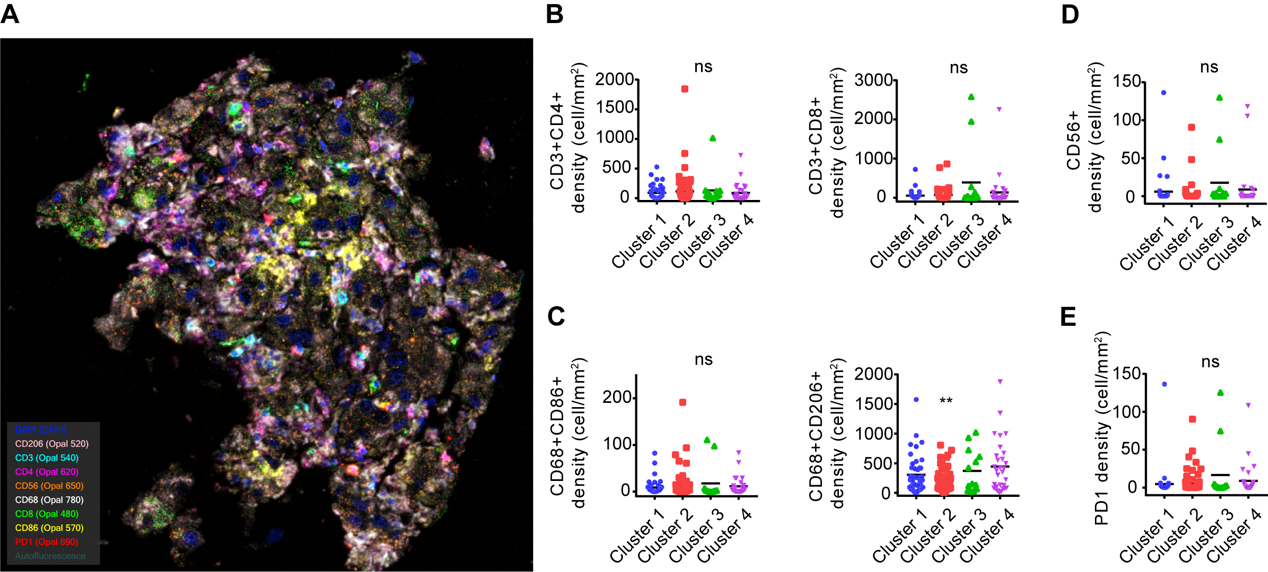
**

**Figure S8. Immune characteristics of metabolic clusters in tumor-adjacent tissues in the ZS-HCC cohort.** (A) Representative multispectral images of normal samples on tissue microarrays of the four clusters in the ZS-HCC cohort. DAPI: cyan; CD3: blue; CD4: purple; CD8: green; CD68: white, CD86: yellow; CD20: pink; CD56: orange; and PD-1: red. (B-E) Distribution of different makers in the four clusters of the ZS-HCC cohort. (B) CD3+CD4+ and CD3+CD8+; (C) CD68+CD86+ and CD68+CD206+; (D) CD56+; (E) PD-1. Data are presented as the mean ± SD; Tukey’s post hoc test. ** *P* < 0.01; ns *P* > 0.05.

**
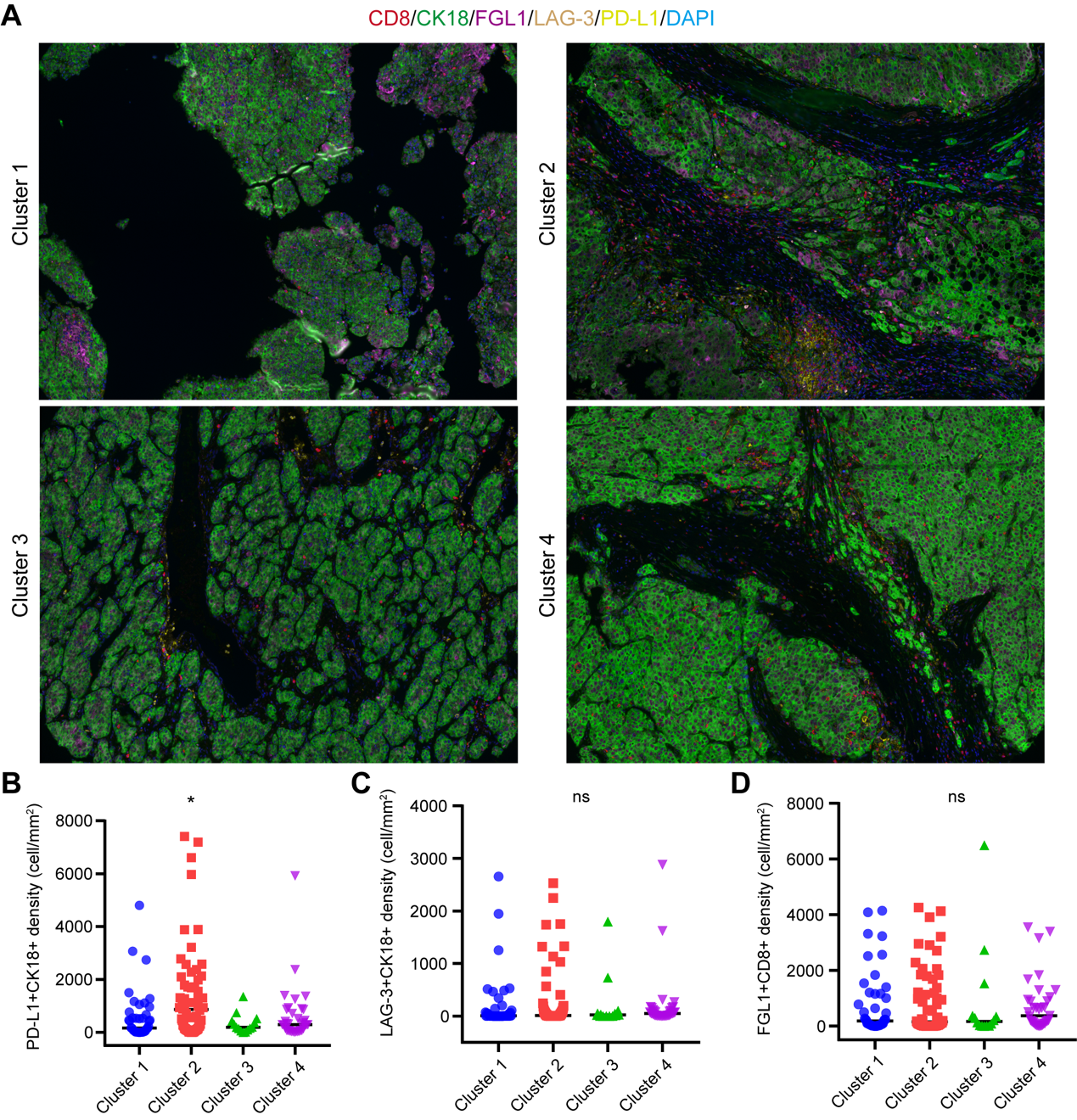
**

**Figure S9. Characteristics of three immune checkpoints (FGL1, LAG3, and PD-L1) of metabolic clusters in the ZS-HCC cohort.** (A) Representative multispectral images of HCC samples on tissue microarrays of the four clusters in the ZS-HCC cohort. DAPI: cyan; CD8: red; CK18: green; FGL1: purple; LAG-3: orange; and PD-L1: yellow. (B-D) Distribution of PD-L1 (B), LAG-3 (C) and FGL1 (D) expression in the four clusters in the ZS-HCC cohort. Data are presented as the mean ± SD; Tukey’s post hoc test. **P* < 0.05; ns *P* > 0.05.


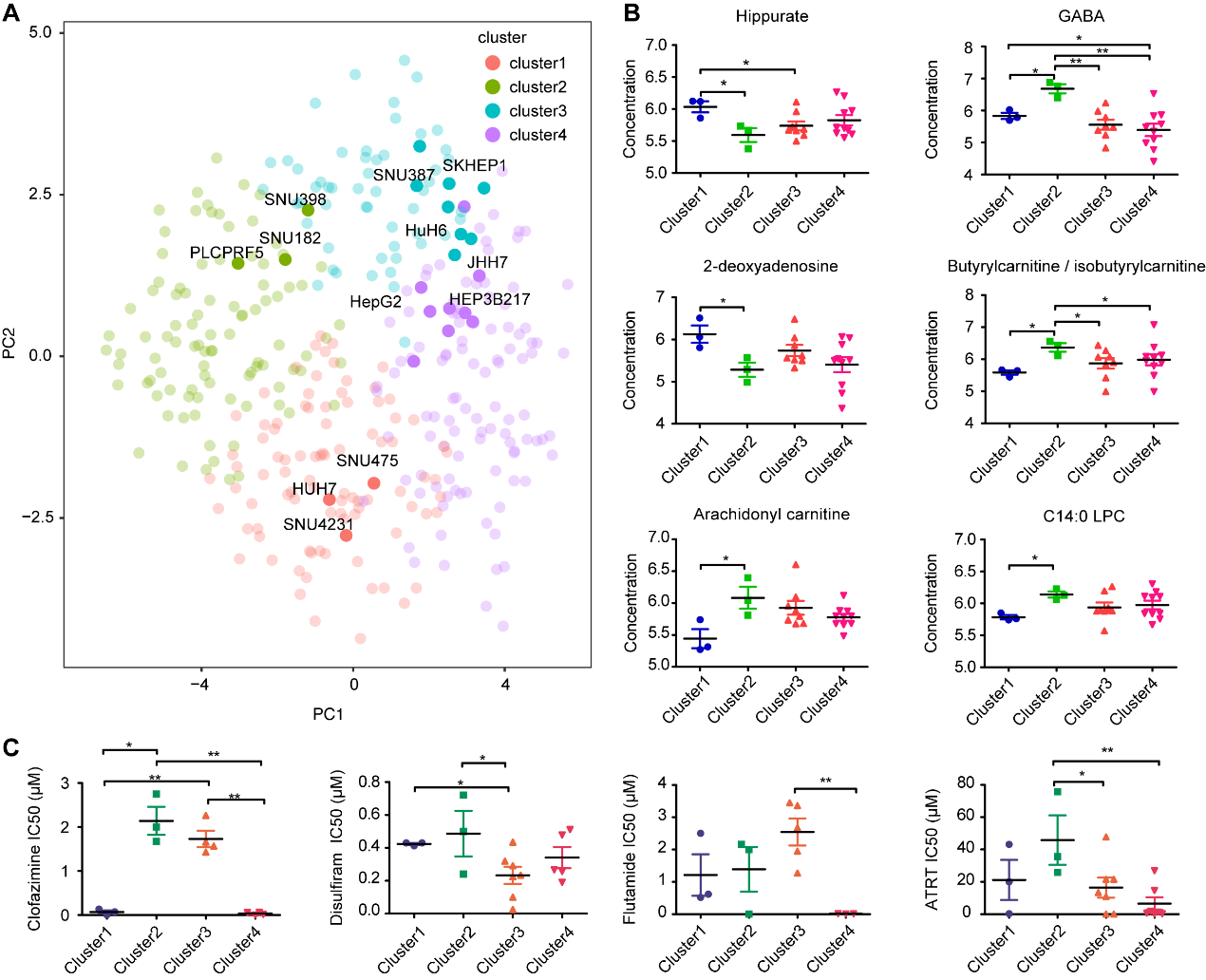


**Figure S10. Metabolic clusters of 12 human HCC cell lines from the Cancer Cell Line Encyclopedia.** (A) The nearest shrunken centroids method was used to classified 12 human HCC cell lines into four clusters. (B) Relative intensity levels of metabolites involved in carbohydrate metabolism, lipid metabolism and amino acid metabolism were differentially expressed among the four metabolic clusters of HCC cell lines. (C) Drug sensitivity of the four metabolic clusters of HCC cell lines. The Y-axis represents half maximal inhibitory concentration of drugs, including clofazimine, disulfiram, flutamide and ATRA. Data are presented as the mean ± SD; Tukey’s post hoc test. ***P* < 0.01; **P* < 0.05.


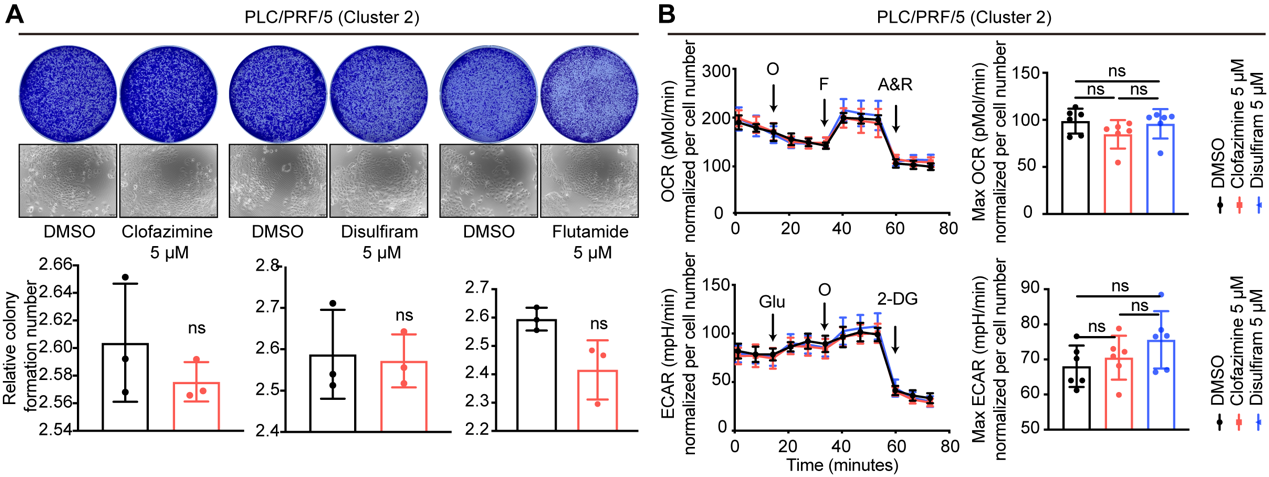


**Figure S11. Effects of metabolic drugs in PLC/PRF/5.** (A) Representative pictures of colony formation assays and quantification of colony formation assays with different drug treatment in PLC/PRF/5. Data are presented as the mean ± SD of 3 independent experiments; Student’s test. ns *P* > 0.05. (B) Oxygen consumption rate (OCR) and extracellular acidification rate (ECAR) levels were measured using the Seahorse assay, and the basal/maximal respiration and glycolysis levels were calculated accordingly with different drug treatment in PLC/PRF/5. Data are shown as the mean ± SD of 6 independent experiments. Tukey’s post hoc test. ns *P* > 0.05.


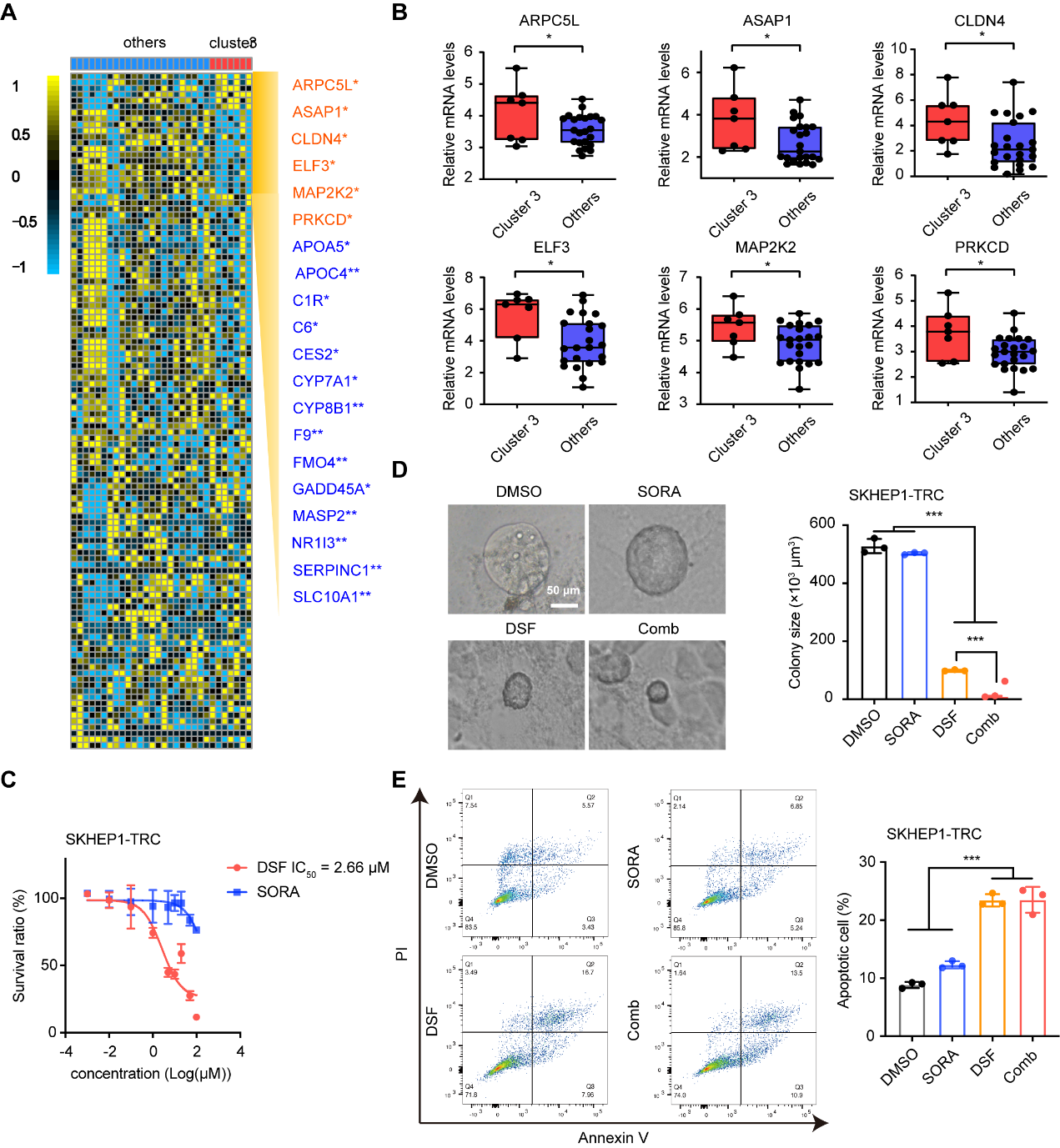


**Figure S12. Characteristics of** **stemness associated genes among metabolic clusters in the ZS-SEQ-HCC cohort.** (A) Heatmap shows relative expression of stemness associated genes between cluster 3 and other clusters in ZS-SEQ-HCC cohort. Genes dysregulated in cluster 3 are shown with asterisk. Student’s t test. ***P* < 0.01; **P* < 0.05. (B) Boxplots show relative mRNA expression of key stemness associated genes in cluster 3 and other clusters in the ZS-SEQ-HCC cohort. Data are presented as the mean ± SD; Student’s t test. **P* < 0.05. (C) Effects of disulfiram or sorafenib treatment on HCC (SKHEP1) -TRC. (D) Effects of disulfiram treatment on colony spheroid formation and sensitivity to sorafenib of HCC (SKHEP1) -TRC. (E) Effects of disulfiram treatment on cell apoptosis and sensitivity to sorafenib of HCC (SKHEP1) -TRC. Data are shown as the mean ± SD of 3 independent experiments. Tukey’s post hoc test. ****P* < 0.001.
